# Disabled-2: a positive regulator of the early differentiation of myoblasts

**DOI:** 10.1007/s00441-020-03237-2

**Published:** 2020-06-30

**Authors:** Na Shang, Juliana Tsz Yan Lee, Taida Huang, Chengdong Wang, Tin Lap Lee, Samuel C. Mok, Hui Zhao, Wood Yee Chan

**Affiliations:** 1 School of Biomedical Sciences, Faculty of Medicine, The Chinese University of Hong Kong, Lo Kwee-Seong Integrated Biomedical Sciences Building, Hong Kong SAR, China; 2 Division of Surgery, Department of Gynecologic Oncology and Reproductive Medicine, The University of Texas M.D. Anderson Cancer Center, T4.3908, 1515 Holcombe Boulevard, Houston, TX 77030 USA

**Keywords:** *Disabled* gene, Skeletal myogenesis, C2C12 cells, *Xenopus laevis* embryos, Mef2c

## Abstract

Dab2 is an adaptor protein and a tumor suppressor. Our previous study has found that *Dab2* was expressed in early differentiating skeletal muscles in mouse embryos. In this study, we determined the role of Dab2 in the skeletal muscle differentiation using C2C12 myoblasts in vitro and *Xenopus laevis* embryos in vivo. The expression of *Dab2* was increased in C2C12 myoblasts during the formation of myotubes in vitro. Knockdown of *Dab2* expression in C2C12 myoblasts resulted in a reduction of myotube formation, whereas the myotube formation was enhanced upon overexpression of *Dab2*. Re-expression of *Dab2* in C2C12 myoblasts with downregulated expression of *Dab2* restored their capacity to form myotubes. Microarray profiling and subsequent network analyses on the 155 differentially expressed genes after *Dab2* knockdown showed that Mef2c was an important myogenic transcription factor regulated by Dab2 through the p38 MAPK pathway. It was also involved in other pathways that are associated with muscular development and functions. In *Xenopus* embryos developed in vivo, *XDab2* was expressed in the myotome of somites where various myogenic markers were also expressed. Knockdown of *XDab2* expression with antisense morpholinos downregulated the expression of myogenic markers in somites. In conclusion, this study is the first to provide solid evidence to show that Dab2 is a positive regulator of the early myoblast differentiation.

**Electronic supplementary material:**

The online version of this article (10.1007/s00441-020-03237-2) contains supplementary material, which is available to authorized users.

## Introduction

DAB2 was also named DOC-2, which is differentially expressed in human ovarian carcinoma cells (Mok et al. 1994, 1998). It was then identified as one of two mammalian orthologs of *Drosophila* Disabled (dDab) (Xu et al. 1995; Sheng et al. 2000, 2001). In the mouse, *Dab2* has three splice isoforms, encoding p96, p93, and p67 proteins, among which p96 is the main isoform (Xu et al. 1995; Sheng et al. 2000). Dab2 proteins show features of cytoplasmic adaptor proteins which contain protein-binding domains and phosphorylation sites but do not have catalytic regions (Xu et al. 1995; Howell et al. 1997), and therefore, they are potentially able to participate in different signaling pathways (Pawson and Scott 1997). *Dab2* is expressed in different carcinomas and characterized as a tumor suppressor (Mok et al. 1994; Schwahn and Medina 1998; Tseng et al. 1998; Huang et al. 2001). It has also been found in a multitude of tissues and cell types including primitive endodermal cells (Yang et al. 2002), mesoderm-derived cells such as human K562 cells (Tseng et al. 2003), endothelial cells (Cheong et al. 2006), zona glomerulosa of the adrenal cortex (Romero et al. 2007), bone marrow-derived macrophages and lymphocytes (Rosenbauer et al. 2002; Jain et al. 2009), and embryonic stem cells (Huang et al. 2010). The functions of Dab2 in these tissues and cells have been linked to the regulation of endodermal cell organization, cell adhesion, cell spreading and differentiation, blood vessel formation, aldosterone secretion, and mesoderm differentiation.

Skeletal myogenesis is a complex cascade of events that involve the specification and differentiation of muscle precursor cells or myoblasts, fusion of myoblasts to form primary and secondary myotubes, and their subsequent maturation into myofibers (Charge and Rudnicki 2004). Various transcription factors which have been found to modulate the differentiation of muscle cells include the paired box transcription factors Pax7 and Pax3, the myogenic regulatory factors (MRFs), and the myocyte enhancer–binding factor 2s (MEF2s). MRFs consist of four basic helix-loop-helix (bHLH) proteins (Myf5, MyoD, myogenin, and MRF4), each heterodimerizing with E proteins (E12 or E47) when binding to the E box (CANNTG) in the promoter of many muscle-specific genes (Tapscott 2005). MEF2s have four MADS-box-containing proteins (MEF2A, 2B, 2C, and 2D) that are capable of forming both homo- and heterodimers via consensus A/T-rich DNA sequences present in the regulatory region of many muscle-specific genes (Black and Olson 1998; Naya and Olson 1999; Wang et al. 2008a). This dimerization process is required not only for the development of skeletal muscles but also for the postnatal growth and regeneration of myofibers after injury (Perry and Rudnick 2000; Charge and Rudnicki 2004).

In our previous study on the expression of *Dab2* in the developing central nervous system (Cheung et al. 2008), strong immunoreactivities were localized to somites and skeletal muscles of mouse embryos. Another study also showed that *XDab2* was expressed in somites of *Xenopus* embryos (Cheong et al. 2006). Somites are segmental derivatives of the paraxial mesoderm in vertebrate embryos and are one of the embryonic origins of skeletal muscles and satellite cells (Christ and Ordahl 1995; Gros et al. 2005; Schienda et al. 2006). In a study with microarrays, the expression of *Dab2* was upregulated in C2C12 myoblasts when they were induced to differentiate in vitro (Tomczak et al. 2004). It was also observed that a higher incidence of runt neonates was born to heterozygous *Dab2* mutant mice although these heterozygous mice appeared to be overtly normal (Yang et al. 2002). This retarded development has been ascribed to the abnormal development of the musculoskeletal system. In our studies, Dab2 was also found to co-localize with two important transcription factors Pax3 and Myf5 during muscle development, and changes in Dab2 expression were associated with alterations of myotube formation (Shang et al. 2011). However, the exact roles of Dab2 in the skeletal muscle differentiation into myofibers and the molecules that are downstream of Dab2 in the regulation of skeletal muscle development have not been fully investigated.

C2C12 myoblasts have been widely used for studies of myogenesis and cell differentiation in vitro (Blau et al. 1985). When cultured in a medium low in serum, C2C12 myoblasts start to differentiate rapidly and form extensive contracting myotubes expressing characteristic muscle proteins. In the present study, with C2C12 myoblasts and *Xenopus* embryos as in vitro and in vivo experimental tools, respectively, we provided the evidence for the first time that the adaptor protein Dab2 is a positive regulator of skeletal muscle differentiation and its orthologue in *Xenopus* (XDab2) is also involved in the somite development.

## Materials and methods

### Cell culture, transfection, and differentiation

C2C12 mouse myoblasts were obtained from the American Type Culture Collection (ATCC) and maintained in the growth medium consisting of Dulbecco’s modified Eagle medium (DMEM, Gibco) with 10% (v/v) fetal bovine serum (Biosera). Cell cultures were maintained in the absence of antibiotics except for the medium used for the culture of stable transfected cell lines which contained 2 μg/ml puromycin (Sigma). Lipofectamine™ 2000 (Invitrogen) was used for the transient transfection based on the manufacturer’s protocol.

### RNA and protein sample preparation

For RNA extraction, C2C12 myoblasts were cultured in 60-mm dishes, washed with ice-cold PBS twice, and lysed in the cell lysis buffer from the SV total RNA isolation kit (Promega) according to the manufacturer’s protocol. For protein extraction, cells in 100-mm dishes were washed with ice-cold PBS twice and lysed with the 500-μl RIPA lysis buffer (Cell Signaling) supplemented with 1 mM phenyl-methylsulfonyl fluoride (PMSF, Sigma) and protease inhibitor cocktail (Roche). The cell lysates detached by a cell scraper were then centrifuged at 4 °C with 15,000 rpm for 15 min, and the supernatants were stored at − 80 °C.

### C2C12 differentiation in vitro and immunofluorescence staining

C2C12 cells were induced to differentiate by changing the growth medium to the differentiation medium (DMEM containing 2% normal horse serum (NHS, Gibco)) which was replenished every 2 days (Tomczak et al. 2004). DMEM containing 2% NHS supplemented with 10 μM insulin (I1882, Sigma) was used for the studies of myotube formation. For immunofluorescence staining, cells growing on gelatin-coated coverslips were fixed in 4% paraformaldehyde for 15 min. The standard methods for immunofluorescence staining were used as previously described (Mok et al. 1998), and the antibodies used in the study are listed in Supplementary Table 1. After staining, cells were finally mounted with 75% glycerol in phosphate buffered saline (pH 7.4, 0.01 M) containing 1.5 μg/ml 4′,6′-diamidino-2-phenylindole hydrochloride (DAPI). Images were taken under a confocal microscope (Olympus FV1000). The myogenic fusion index was calculated as previously described (Sun et al. 2010).

### Plasmid construction

The miRNA plasmids for silencing *Dab2* (pcDNA™6.2-GW/±EmGFP-miR-Dab2) were constructed by following the protocol from the kit (K4936-00, Invitrogen). Human *DAB2* (EX-M0885-lv105, GeneCopoeia), mouse *Dab2* short isoform (p67) (EX-Mm02067-lv105, GeneCopoeia), mouse *Dab2* long isoform (p96) (EX-Mm24887-lv105, GeneCopoeia) expression plasmids, and control plasmid (EX-NEG-lv105, GeneCopoeia) were used. The *Mef2c* expression plasmid was constructed by cloning the *Mef2c* coding sequence (CDS) into the pCS2+ vector and fused with a HA tag. Primers used in the study are listed in Supplementary Table 2.

### Lentiviral shRNA mediated *Dab2* silencing and stable cell line generation

MISSION™ shRNA lentiviral transduction particles including a nontargeting shRNA control (Sigma) were used for generating *Dab2* stable knockdown in C2C12 myoblasts (Supplementary Table 2). Lentiviral supernatant containing viruses was spin-infected (2250 rpm, 60 min at room temperature) using polybrene (8 μg/ml) to C2C12 cells (6000–8000 cells/well in 96-well plates) at multiplicity of infection (MOI) of 5. For stable cell line generation, the transfected C2C12 cells were subcultured and diluted to no more than one cell in each well of a 96-well plate. The cells were maintained in the growth medium containing 2 μg/μl puromycin for selecting positive infection. Cell colonies were formed in about 4–6 days after integration of lentiviral shRNA into the genome. The colonies were expanded, examined by real-time PCR for knockdown efficiency, and cryo-preserved in liquid nitrogen for further use.

### Serum starvation and FGF treatment of C2C12 cells

Prior to the fibroblast growth factor (FGF) treatment, cells in 100-mm dishes with 80% confluence were washed with serum-free DMEM twice and maintained in DMEM without serum for 8 h. Then, serum-starved cells were treated with serum-free DMEM containing 1 nM FGF. At designated time points (0 min, 5 min, 15 min, 30 min, 60 min, 90 min, and 120 min), cells were harvested with the protein lysis buffer as mentioned previously and followed by western blotting for the expression of p38 MAPK and p-p38 MAPK.

### Microarray and data analysis

For profiling gene expression, total RNA from clone 5-2 (C2C12 cells with stable knockdown of *Dab2*) and control C2C12 cells (transfected with nontargeting shRNA), both of which were cultured in the differentiation medium for 2 days, were extracted as mentioned above previously. GeneChip® Mouse Gene 1.0 ST Arrays (Affymetrix, 901168) were used following the manufacturer’s instruction. The raw data of the microarrays were imported to the Partek Genomics Suite (version 6.4, Partek Inc., St. Louis, MO) for probe set summarization and statistical analysis. Analysis of variance (ANOVA) was used to calculate the *p* values so as to determine statistically significant differences in the mean transcript abundance for each probe set between C2C12 cells (*n* = 2) and clone 5-2 (*n* = 2). A *p* value of less than 0.05 was defined as statistically significant. The integrated gene network with significant changes in expression was generated by Ingenuity Pathways Analysis (IPA; Ingenuity Systems, www.ingenuity.com).

### Frog embryo microinjection and in situ hybridization

*Xenopus laevis* eggs were fertilized in vitro (Sive et al. 2007) and embryos were staged as described (Keller 1991). The splicing morpholinos (Genetools) against *Xenopus Dab2* (*XDab2*) were constructed as described previously (Cheong et al. 2006). Microinjection, whole-mount in situ hybridization, and vibratome sectioning were performed as described in our previous studies (Kam et al. 2010; Wang et al. 2010). Phylogenetic and quantitative PCR (qPCR) analyses were performed as described previously (Wang et al. 2010). The primers for qPCR are listed in Supplementary Table 3.

*Xenopus laevis* embryos were hybridized with probes for *XDab2* (Cheong et al. 2006), *XPax3* (Zhao et al. 2008), *XMyos* (Zhao et al. 2007), *XMyoD* (Fisher et al. 2002), *Xac100* (Hemmati-Brivanlou et al. 1990), and *XMyf-5* (Dosch et al. 1997). *XDab2L* and *XMef2c* probes were prepared by subcloning their targeting sequences into the pCS2+ and pBluescript II KS (+/−) vectors, respectively, and plasmids were linearized with HindIII and XhoI accordingly for preparing probe templates. The digoxigenin-labeled antisense probes were synthesized with T7 RNA polymerase from Roche Dig-RNA Labeling Kit (Roche).

### Statistical analyses

All statistical results were presented as mean ± standard deviation (SD). Paired two-tailed Student’s *t* test and one-way ANOVA were used with a confidence level at 95%. *p* < 0.05 was considered statistically significant.

## Results

### *Dab2* expression was upregulated upon myogenic differentiation and downregulated after myotube formation

**Fig. 1:**
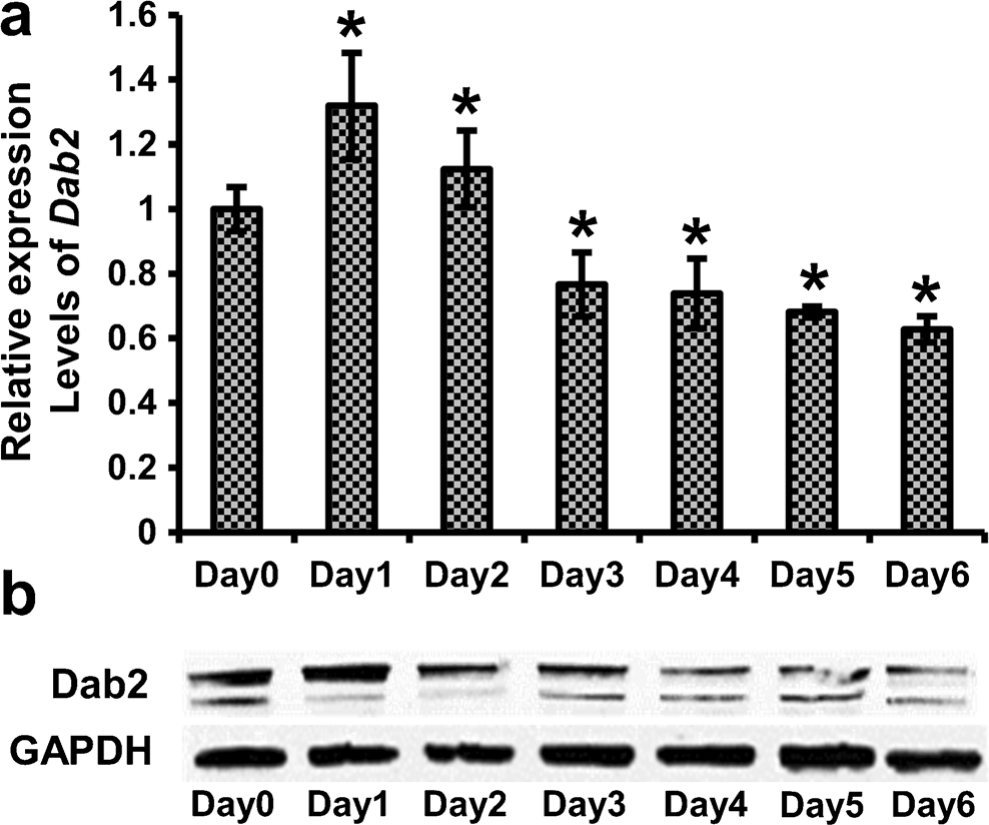
Expression of *Dab2* mRNAs and Dab2 proteins during C2C12 differentiation. C2C12 cells at 80% confluence were induced to differentiate with the differentiation medium containing 2% normal horse serum (NHS) on day 0, and harvested at different time points for (**a**) qPCR and (**b**) western blot. Anti-Dab2 antibodies are able to recognize all three isoforms. *p < 0.05 (*n* ≥ 3) significantly different from the value on day 0, one-way ANOVA followed by Turkey’s test. Data are expressed as mean ± SD. Expression levels of *Dab2* mRNA are normalized against the *Gapdh* level

The C2C12 muscle cells are a stable myoblast cell line with constant levels of Dab2 expression (for basal levels of Dab2 expression, see day 0 group in Fig. 1a, b and the control transfection group from 24 to 48 h in Fig. 3(a)) and were used to investigate the potential roles of Dab2 in the myogenic differentiation in vitro. On days 1 and 2 after induction of C2C12 cell differentiation with the differentiation medium containing 2% normal horse serum (NHS), the expression of *Dab2* mRNA was significantly increased (*n* ≥ 3) (Fig. 1a). On days 3 to 6, after myotubes were formed, the expression of *Dab2* became downregulated (Fig. 1a). Consistently, western blot analysis (Fig. 1b) also showed that the protein level of Dab2 was high on days 1 and 2, and then started to decline from day 3. These observations indicate the potential role of Dab2 in promoting the early differentiation of myoblasts.

### Downregulated expression of *Dab2* suppressed myogenic differentiation

**Fig. 2:**
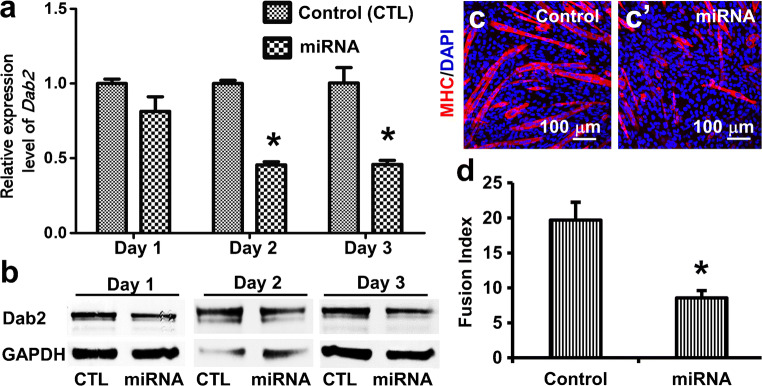
Knockdown of *Dab2* led to decreased myogenic differentiation. (a, b) C2C12 cells were transfected with control or *Dab2* miRNA plasmids, and cells harvested on days 1, 2, and 3 after transfection were further processed for (a) qPCR (*n* = 3) and (b) western blot (*n* = 3). Anti-Dab2 antibodies are able to recognize all three isoforms. *p < 0.01, Student’s *t* test. (c) On day 3 post-transfection when cells reached about 80% confluence, they were induced to differentiate with the differentiation medium containing 2% NHS for 5 more days. Then the cultures were immunofluorescently stained with an antibody against MHC, a marker of myotubes, and cell nuclei were stained with DAPI. Three independent experiments were performed, and similar outcomes were obtained. Representative photomicrographs are shown. (d) Bar chart showing myogenic fusion indices of the control and miRNA groups. The myogenic fusion indices were calculated as described under the “Materials and methods.” *p < 0.01 (*n* ≥ 4), Student’s *t* test. Data are expressed as mean ± SD

To knock down the expression of *Dab2*, we transfected C2C12 cells with *Dab2* miRNA plasmids (Fig. 2). Significant downregulation of *Dab2* expression in the *Dab2-*miRNA transfected cells was observed on days 2 and 3 post-transfection as compared with the cells transfected with the control plasmid (Fig. 2(a)). Dab2 proteins were also reduced in the knockdown cells on day 3 post-transfection (Fig. 2(b)). C2C12 cells were then induced to differentiate into myotubes on day 3 post-transfection. The *Dab2* knockdown group showed a decreased number of MHC (a marker of myotubes) immunoreactive cells 5 days after induction of differentiation compared to the control group (Fig. 2(c)). A significantly lower myogenic fusion index was also observed in the *Dab2*-miRNA transfected cells than in the cells transfected with the control plasmid (8.6 ± 1.0 vs 19.7 ± 2.5; *n* ≥ 4) (Fig. 2(d)). Thus, Dab2 appears to be essential for myotube formation, and downregulation of *Dab2* expression suppresses myogenic differentiation.

### Upregulation of *Dab2* expression by transient transfection enhanced myogenic differentiation

**Fig. 3:**
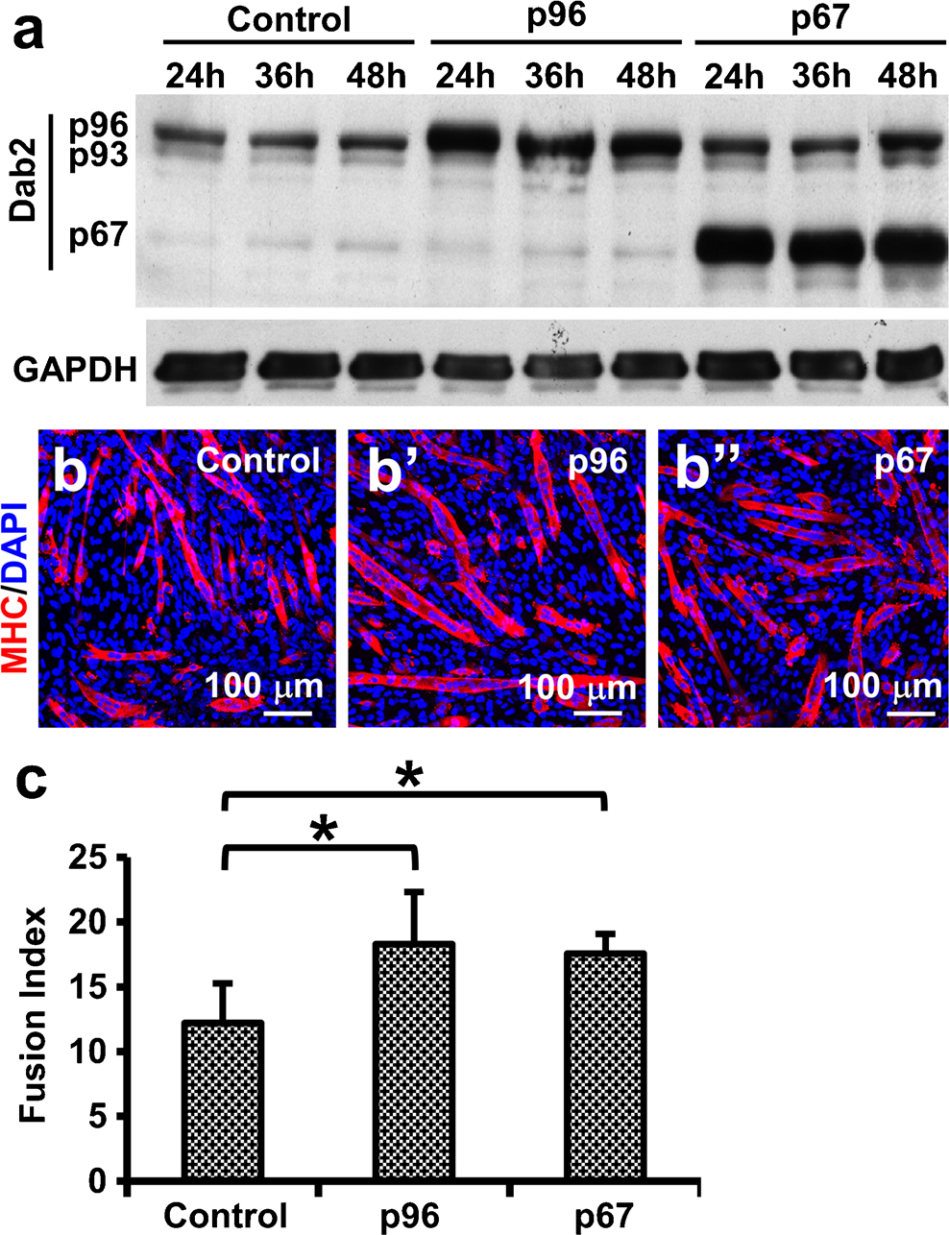
*Dab*2 overexpression led to enhanced myogenic differentiation. C2C12 cells were transfected with the Dab2 expression plasmids (p96 or p67 isoform) or the control plasmid. (a) Western blot showing the expression of Dab2 proteins at 24, 36, and 48 h post-transfection. (b) Representative photomicrographs showing immunoreactivity of MHC in myotubes 5 days after the induction of myogenic differentiation. C2C12 cells were induced to differentiate on day 3 post-transfection when cells were about 80% confluent. (c) Bar chart showing myogenic fusion indices. *p < 0.05 (*n* ≥ 4), significantly different from the control value, Student’s *t* test. Data are expressed as mean ± SD

To further determine the role of Dab2 in early myogenesis, p96 and p67 isoforms of mouse Dab2 were first transiently overexpressed in C2C12 cells separately by transfection of the corresponding Dab2 expression plasmid (Fig. 3(a)), and then on day 3 post-transfection, cells were induced to differentiate. Upregulation of either p96 or p67 isoform enhanced myotube formation 5 days after induction of differentiation (Fig. 3(b)). The myogenic fusion index of the p96- (18.3 ± 4.0, *p* < 0.05) or p67- (17.6 ± 1.5, *p* < 0.05) transfected groups was significantly higher than that of the control group (12.2 ± 3.6) (Fig. 3(c)), indicating the positive effect of Dab2 on myotube formation during early myogenic differentiation.

### Lentivirus-mediated *Dab2* stable knockdown reduced myotube formation and affected the p38 MAPK signaling pathway

**Fig. 4:**
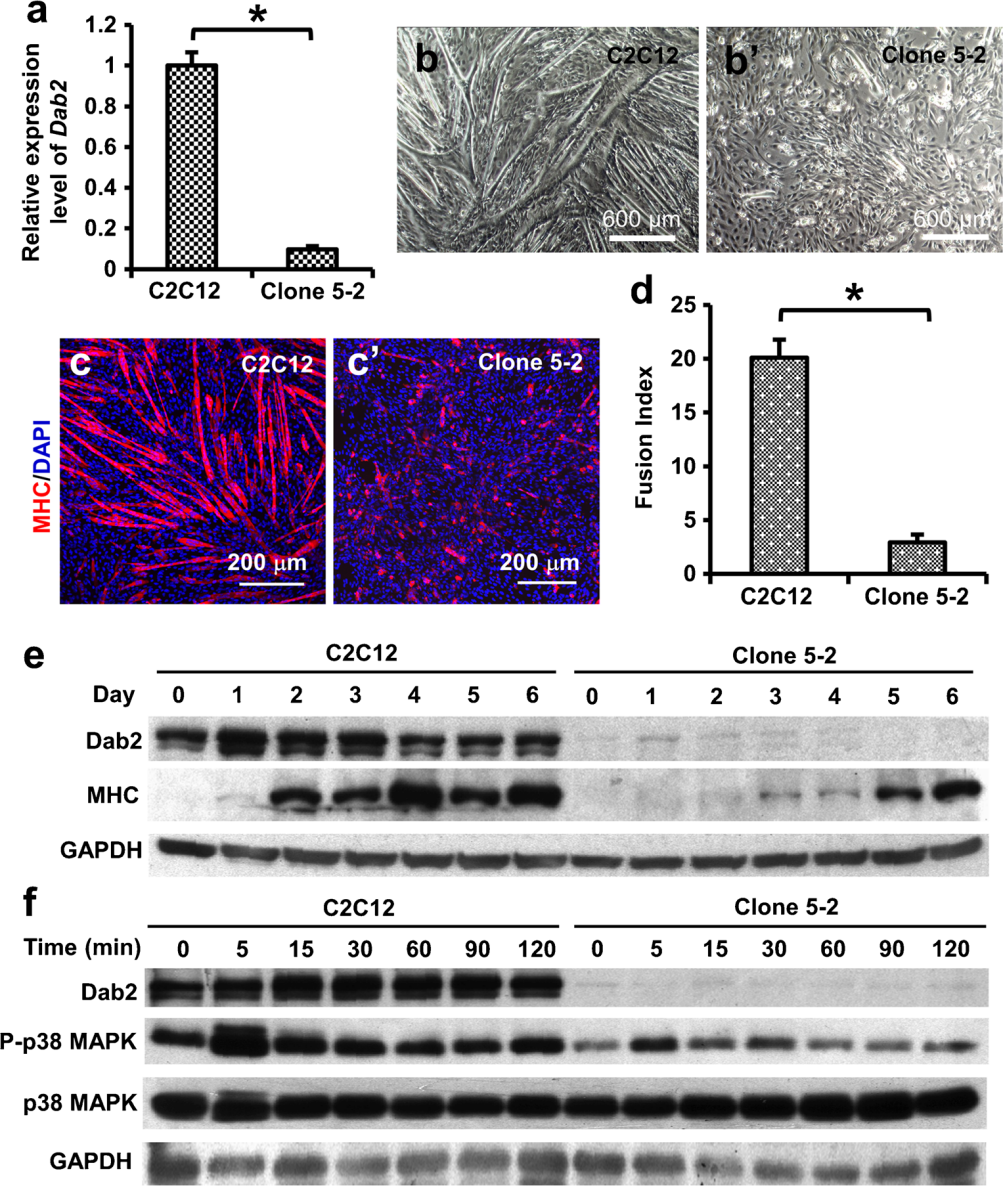
Dab2 stable knockdown reduced myogenic differentiation and phospho-p38 MAPK. C2C12 cells were infected with lentiviruses carrying *Dab2* shRNA at MOI of 5. Clone 5-2 stably expressing *Dab2* shRNA was established in the culture medium containing 2 μg/ml puromycin. (a) qPCR results showing 90.3% *Dab2* knockdown efficiency in clone 5-2 as compared with C2C12 cells in the control group which were infected with nontargeting shRNAs. (b) On day 3 post-infection, control C2C12 cells and clone 5-2 with about 80% confluence were induced to differentiate into myotubes in the differentiation medium containing 2% NHS for 5 days. Representative phase-contrast images are shown. (c) Representative photomicrographs showing myotubes expressing MHC. (d) Bar chart showing myogenic fusion indices. *p < 0.01, *n* = 4, Student’s *t* test. Data are expressed as mean ± SD. (e) Western blot showing expression of Dab2 (p96) and MHC proteins on days 0 to 6 after the induction of differentiation. (f) Western blot showing expression of Dab2 (p96), phospho-p38 MAPK, and p38 MAPK at different time points after the FGF treatment. GAPDH is the loading control

To further confirm the role of Dab2 in early myogenic differentiation and determine the signaling pathway involved, C2C12 cells with *Dab2* stable knockdown were generated using lentiviral shRNA infection. One representative clone named clone 5-2 was selected with puromycin, and the knockdown efficiency for the clone was estimated to be over 90% compared with that observed in control C2C12 cells infected by nontargeting shRNAs (Fig. 4(a)). On day 3 post-infection, myogenic differentiation was induced, and 5 more days later after the induction of differentiation, significantly fewer myotubes were formed in clone 5-2 as compared with those observed in the control C2C12 group (Fig. 4(b)). Immunofluorescence staining showed fewer MHC immunoreactive cells (Fig. 4(c)), as well as a lower fusion index in clone 5-2 than in control C2C12 cells 5 days after the induction of differentiation (Fig. 4(d)). Western blot confirmed the knockdown of Dab2 expression and the concomitant downregulation of MHC immunoreactivities (Fig. 4(e)). Interestingly, the knockdown was extended to all three isoforms of Dab2. These results were consistent with the observations described above in which transient downregulation of *Dab2* expression reduced myotube formation, lending more evidence to show that Dab2 is a positive regulator of myogenic differentiation.

It has long been known that the p38 mitogen-activated protein kinase (p38 MAPK) pathway plays important roles in muscle development (Han et al. 1997), and therefore, it is pertinent to ascertain whether *Dab2* knockdown also affects the p38 MAPK pathway. It has been known that stable cell lines express low levels of phosphorylated p38 under serum starvation, and upon FGF treatment, the p38 MAPK pathway is activated (Sørensen et al. 2008; Maher 1999). We found that after the p38 MAPK pathway was activated by FGF, the expression of p38 MAPK remained unchanged for at least 120 min in both control C2C12 cells and clone 5-2 (Fig. 4(f)). However, the phospho-p38 MAPK expression in control C2C12 cells was increased 5 min after the FGF treatment, and then stayed at the similar level thereafter, whereas the phospho-p38 MAPK expression levels in clone 5-2 were reduced at all the time points examined when compared to those in control C2C12 cells (Fig. 4(f)). Dab2 expression levels were not affected by the FGF treatment in both control C2C12 cells and clone 5-2 at all the time points examined (Fig. 4(f)).

### Re-expression of Dab2 partially restored myogenic differentiation in clone 5-2

**Fig. 5:**
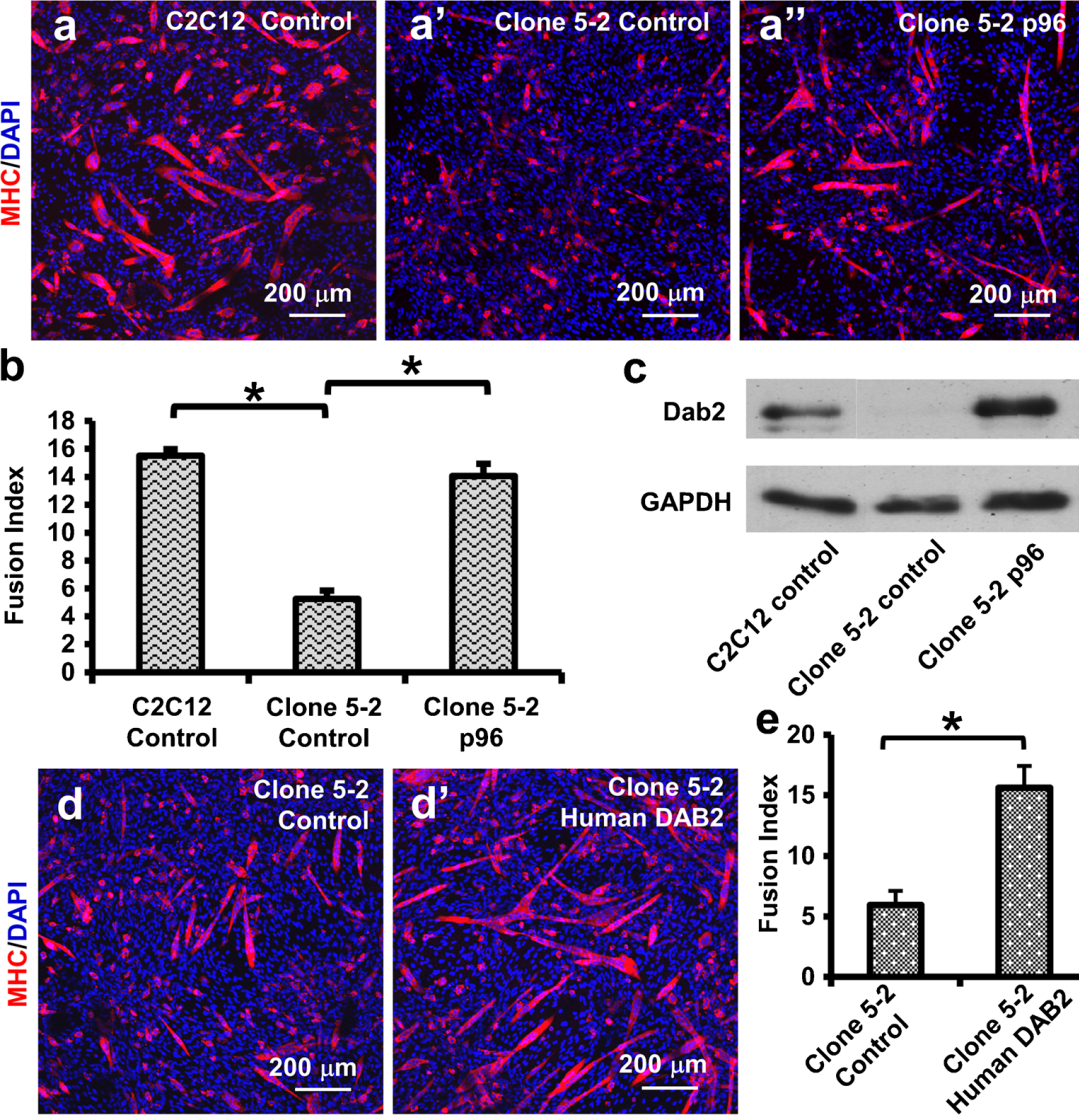
*Dab2* re-expression in clone 5-2 restored myogenic differentiation. Clone 5-2 cells were transfected with the control plasmid (EX-NEG-lv105) (Clone 5-2 Control) or the Dab2 (p96) expression plasmid (Clone 5-2 p96), whereas C2C12 cells transfected with the control plasmid (C2C12 Control) were used as the positive control for myogenic differentiation. On day 3 post-transfection, cells were cultured in the differentiation medium (2% NHS in DMEM) for 5 days to induce myotube formation. Cells were then immunofluorescently stained for MHC, and representative photomicrographs are shown in (a) and myogenic fusion indices shown in (b). (c) Western blot shows that on day 3 post-transfection, *Dab2* expression in clone 5-2 transfected with p96 (Clone 5-2 p96) was restored to the level similar to that of C2C12 cells transfected with the control plasmid (C2C12 Control). (d, e) Clone 5-2 transfected with the human *DAB2* expression plasmid also shows better myogenic differentiation than the clone 5-2 control transfected with the control vector. *p < 0.05, Student’s *t* test, *n* = 4. Data are expressed as mean ± SD

To determine the specificity of the Dab2 knockdown, Dab2 (p96) was re-expressed in clone 5-2 by transfection with the p96 expression plasmid. In the control group, the control vector (EX-NEG-lv105) was transfected to clone 5-2 (i.e., clone 5-2 control group in Fig. 5). On day 3 post-transfection, cells were induced to form myotubes for 5 more days. The reduced formation of myotubes in clone 5-2 was partially restored after Dab2 (p96) was re-expressed (Fig. 5(a)). In clone 5-2 cells transfected with the control vector, the fusion index was found to be 5.2 ± 0.6 (*n* = 6), while in clone 5-2 cells transfected with the p96 expression plasmid, the fusion index was significantly increased to 14.1 ± 0.8 (*n* = 8, *p* < 0.05) which was only 9% lower than that in C2C12 cells transfected with the control vector (15.5 ± 0.5, *n* = 6, no significant difference by Student’s *t* test) (Fig. 5(b)). Western blotting confirmed that Dab2 (p96) was re-expressed in clone 5-2 after transfection with the p96 expression plasmid on day 3 post-transfection (Fig. 5(c)). Interestingly, like mouse *Dab2*, human *DAB2* gene after transfected to clone 5-2 was also able to restore the myogenic differentiation of C2C12 cells in clone 5-2 (Fig. 5(d, e)).

### Network analysis revealed potential functions of Dab2 in C2C12 cells in myogenesis

To further identify the underlying molecular events that led to the phenotypic changes of clone 5-2, a comprehensive profiling of differentially expressed genes in C2C12 cells with and without knockdown of *Dab2* was performed with Affymetrix microarrays. Both control C2C12 and clone 5-2 cells were cultured in the differentiation medium for 2 days before total RNA was extracted for analyses. As compared with the control C2C12 cells, clone 5-2 exhibited significant changes (*p* < 0.05) of 2-folds or greater in the signal intensities of 235 probe sets representing 155 genes, of which 127 were downregulated (Supplementary Table 4) and 28 upregulated (Supplementary Table 5). To verify the observations made with the microarray results, qPCR was performed with the same RNA samples, and results also showed similar changes in the expression of 21 genes (Supplementary Fig. 1) randomly selected from the differentially expressed genes in the microarray (Supplementary Tables 4 and 5). Interestingly, 19 of these 21 genes expressed in clone 5-2 cultured in the proliferation medium also showed similar changes in their expression (Supplementary Fig. 1).

**Fig. 6:**
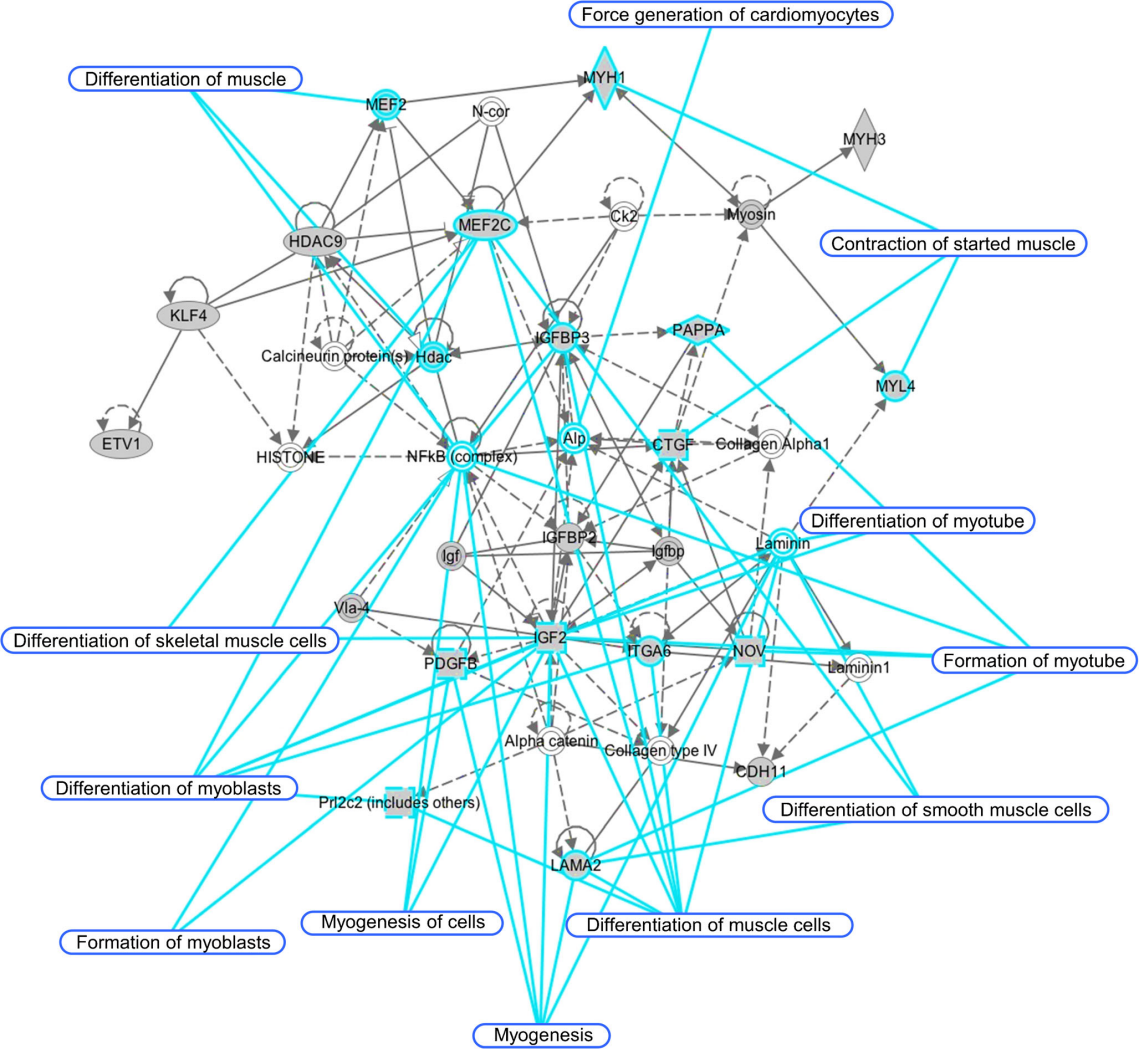
Network analysis of differentially expressed genes in clone 5-2 as compared to control C2C12 myoblasts. Genes whose expression was significantly upregulated or downregulated by 2-folds or more were analyzed by the Ingenuity Pathway Analysis (IPA) software. Representative network that emphasizes the role of *Dab2* in modulating the muscular system is shown. Genes highlighted in gray are identified by microarrays as differentially expressed genes in clone 5-2 with *Dab2* knockdown as compared to control C2C12 myoblasts which were transfected with nontargeting shRNAs

The differentially expressed genes with a change of more than 2-folds in the microarray were analyzed by the Ingenuity Pathway Analysis (IPA). Their biological functions were found to be significantly associated with the development and/or functions of the muscular system. Dab2 potentially affected the expression of genes involved in the contraction of striated muscle, development of muscles, and differentiation of muscle precursor cells (Supplementary Table 6). The expression of some myogenic transcription factors was also changed in clone 5-2 as compared to control C2C12 cells (Supplementary Table 7). When the functional relationships among differentially expressed genes were further analyzed with IPA, a representative network of potential interactions among different molecules related to muscular functions and development was generated (Fig. 6). Mef2c was predicted to be an important hub in this network and has been known to be an important and essential transcription factor regulating myogenic differentiation (Bour et al. 1995; Black and Olson 1998; Wang et al. 2001; Dodou et al. 2003; Haberland et al. 2007). Mef2c was the most affected myogenic transcription factor by *Dab2* knockdown in our microarray analysis. Its expression was significantly decreased by about 3.66-folds in clone 5-2 (Supplementary Table 4), suggesting its important role in the muscular development.

### Re-expression of Mef2c in clone 5-2 restored myotube formation

**Fig. 7:**
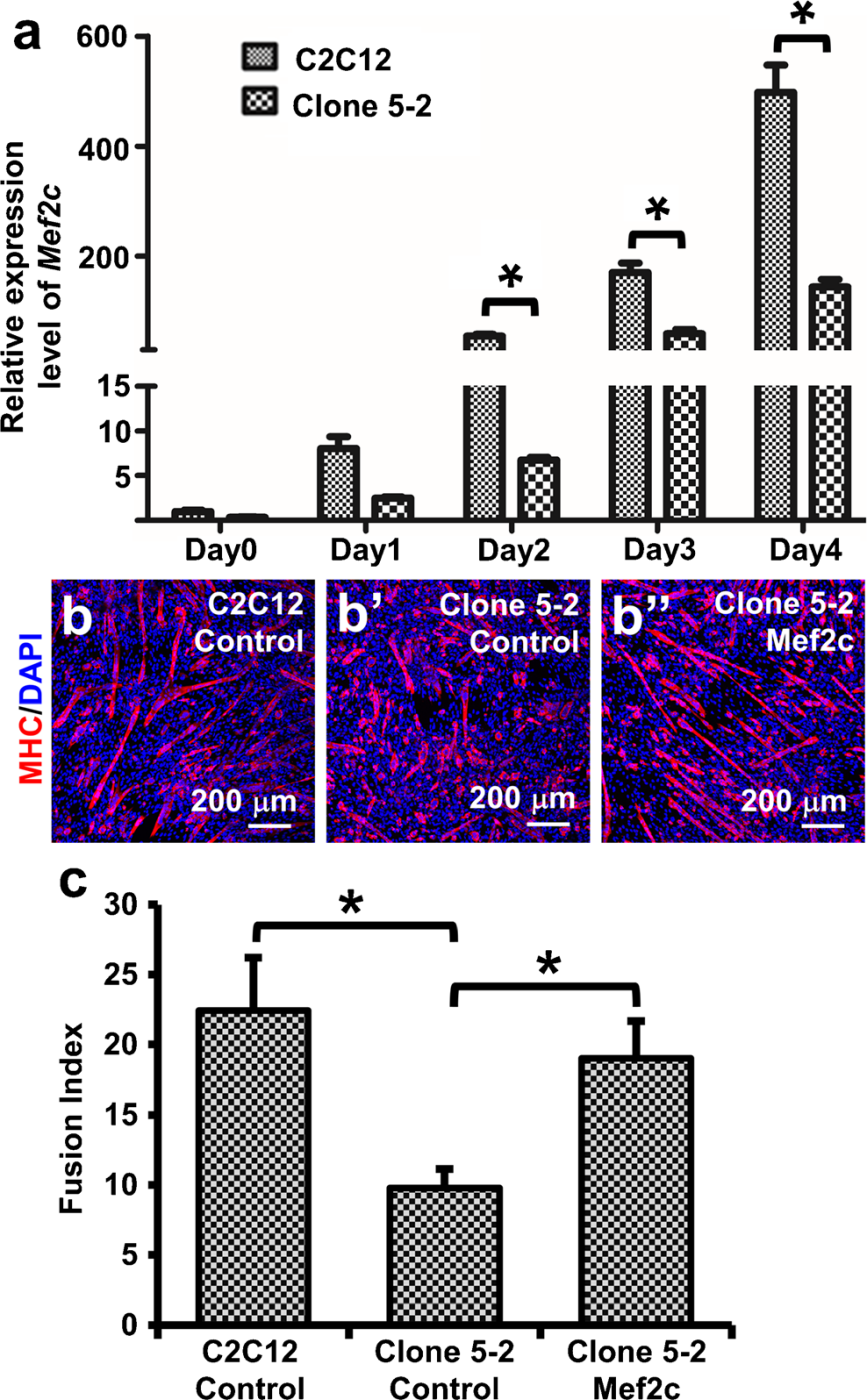
*Mef2c* re-expression in clone 5-2 restored myoblast differentiation. (a) qPCR on the expression of *Mef2c* at different time points after clone 5-2 and C2C12 cells at 80% confluence were induced to differentiate with the differentiation medium containing 2% NHS on day 0. (b) Representative photomicrographs showing MHC immunoreactivities. Clone 5-2 cells were transfected with the control plasmid pCS2+ (Clone 5-2 Control) or the Mef2c expression plasmid (Clone 5-2 Mef2c), and C2C12 cells transfected with the control plasmid (C2C12 Control) serve as the positive control. On day 3 post-transfection, cells were induced to differentiate with the differentiation medium for 5 days, and then immunofluorescently stained for MHC. (c) Bar chart showing myogenic fusion indices 5 days after the induction of myotube formation. *p < 0.05, Student’s *t* test, *n* = 4. Data are expressed as mean ± SD

From the network analysis, we speculated that downregulated expression of *Mef2c* might result in a reduction of myotube formation in clone 5-2 with *Dab2* knockdown. To prove this speculation, we first examined the expression of *Mef2c* by qPCR at different time points after the induction of myogenic differentiation (Fig. 7(a)). The expression of *Mef2c* was significantly reduced in clone 5-2 as compared with the control C2C12 cells from day 1 to day 5. Then, we transfected clone 5-2 cells with the *Mef2c* expression plasmid. On day 3 post-transfection, cells were induced to form myotubes for 5 days. More long myotubes were formed in the *Mef2c*-transfected clone 5-2 cells than the clone 5-2 cells transfected with the control plasmid (pCS2+) only (Fig. 7(b)), and the number and the morphology of the MHC immunoreactive cells in *Mef2c*-transfected clone 5-2 were comparable to those found in C2C12 control cells transfected with the control plasmid (Fig. 7(b)). This observation was confirmed by their corresponding changes in the fusion indices (Fig. 7(c)): the fusion index of the clone 5-2 cells transfected with the pCS2+ control plasmid was only 9.8 ± 1.4 (*n* = 4), whereas that of the *Mef2c*-transfected clone 5-2 cells was significantly increased to 19.0 ± 2.7 (*n* = 4) which was similar to that of the C2C12 control group (22.4 ± 3.8, *p* > 0.5).

### Spatiotemporal expression of *XDab2* long and short isoforms during early embryonic development of *Xenopus laevis*

**Fig. 8:**
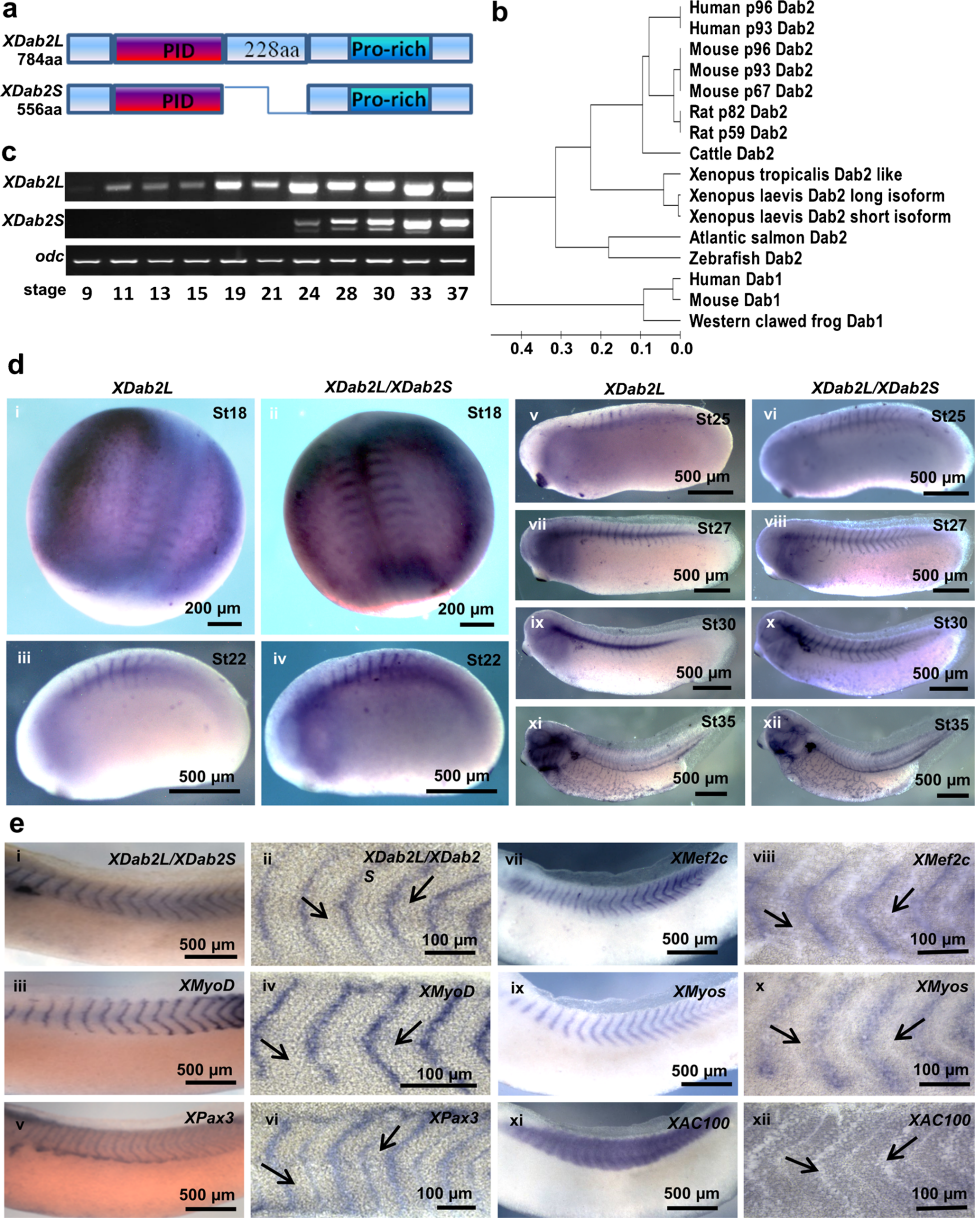
Relationship of *XDab2L* and *XDab2S* and their spatiotemporal expression patterns. (**a**) Schematic representation of different domains of *XDab2L* and *XDab2S*. (**b**) Phylogenetic analysis of Dab1 and Dab2 proteins from the frogs, human, mouse, rat, cattle, and fish. (**c**) qPCR results showing *XDab2L* and *XDab2S* expression at different stages of the *Xenopus laevis* development with ornithine decarboxylase (*odc*) as the internal control. (**d**) Expression of *XDab2L* and *XDab2L/XDab2S* in *Xenopus* embryos at different developmental stages after whole-mount in situ hybridization. (**e**) Vibratome sections showing the expression of *XDab2L/XDab2S* (i, ii), *XMyoD* (iii, iv), *XPax3* (v, vi), *XMef2c* (vii, viii), *XMyos* (ix, x), and *XAC100* (xi, xii) after whole-mount in situ hybridization at stage 30. Boundaries of somites are indicated by arrows

We demonstrated that Dab2 played essential roles in regulating early myogenic differentiation of C2C12 cells in vitro. To further determine its roles in vivo, the expression of *XDab2L* (long isoform) and *XDab2S* (short isoform) (Fig. 8a, b) during the early embryonic development of *Xenopus laevis* was examined by qPCR (Fig. 8c). *XDab2L* expression began at stage 9, while the initial expression of *XDab2S* was observed much later at stage 24. The expression levels of both *XDab2L* and *XDab2S* were increased from the late neurula and tailbud stages.

As *XDab2S* mRNA sequence forms part of the sequence of *XDab2L* (Fig. 8a), probes for in situ hybridization were prepared to examine the expression of both *XDab2L* and *XDab2L/XDab2S* (Fig. 8d). Generally, *XDab2L* and *XDab2L/XDab2S* showed similar spatial expression patterns, and signals of *XDab2L* and *XDab2S* were stronger than those of *XDab2L* alone because probes could bind to the overlapping sequence of *XDab2L* and *XDab2S*. *XDab2L* and *XDab2L/XDab2S* were first expressed in the pre-somitic mesoderm and somites at stage 18 (Fig. 8d (i, ii)), then more intensively in somites at the early tailbud stage (Fig. 8d (iii, iv)), and in the myotomes of somites at stages 25–30 (Fig. 8d (v–x)). At stage 35 (Fig. 8d (xi, xii)), *XDab2L* and *XDab2L/XDab2S* were found to be widely expressed in the vascular system of the embryo with a decrease of expression in somites.

Vibratome sections after whole-mount in situ hybridization at stage 30 (Fig. 8e) showed that *XDab2* was expressed in the myotomes (middle part of somites) (Fig. 8e (i, ii)). Expression of myogenic transcription factors such as *XMyoD* (Fig. 8e (iii, iv)), *XPax3* (Fig. 8e (v, vi)), *XMef2c* (Fig. 8e (vii, viii)), and *XMyos* (Fig. 8e (ix, x)) was also found in the myotomes of somites, where *XDab2* was expressed (Fig. 8e (i, ii)). The expression of a somite marker, *XAC100*, was observed throughout the myotomes of somites (Fig. 8e (xi, xii)). The expression of *XDab2* and all the myogenic transcription factors examined in the myotome plausibly implicated that *XDab2* might have some roles to play in the somite and muscle development.

### Knockdown of *Dab2* led to downregulation of the expression of myogenic transcription factors and a somite marker

To ascertain the role of XDab2 in the somite and skeletal muscle development, antisense morpholino oligonucleotides (MO) was utilized to inhibit the expression of *XDab2* (both long and short isoforms) (*XDab2*-MO1 and *XDab2*-MO2, Supplementary Table 2).

**Fig. 9:**
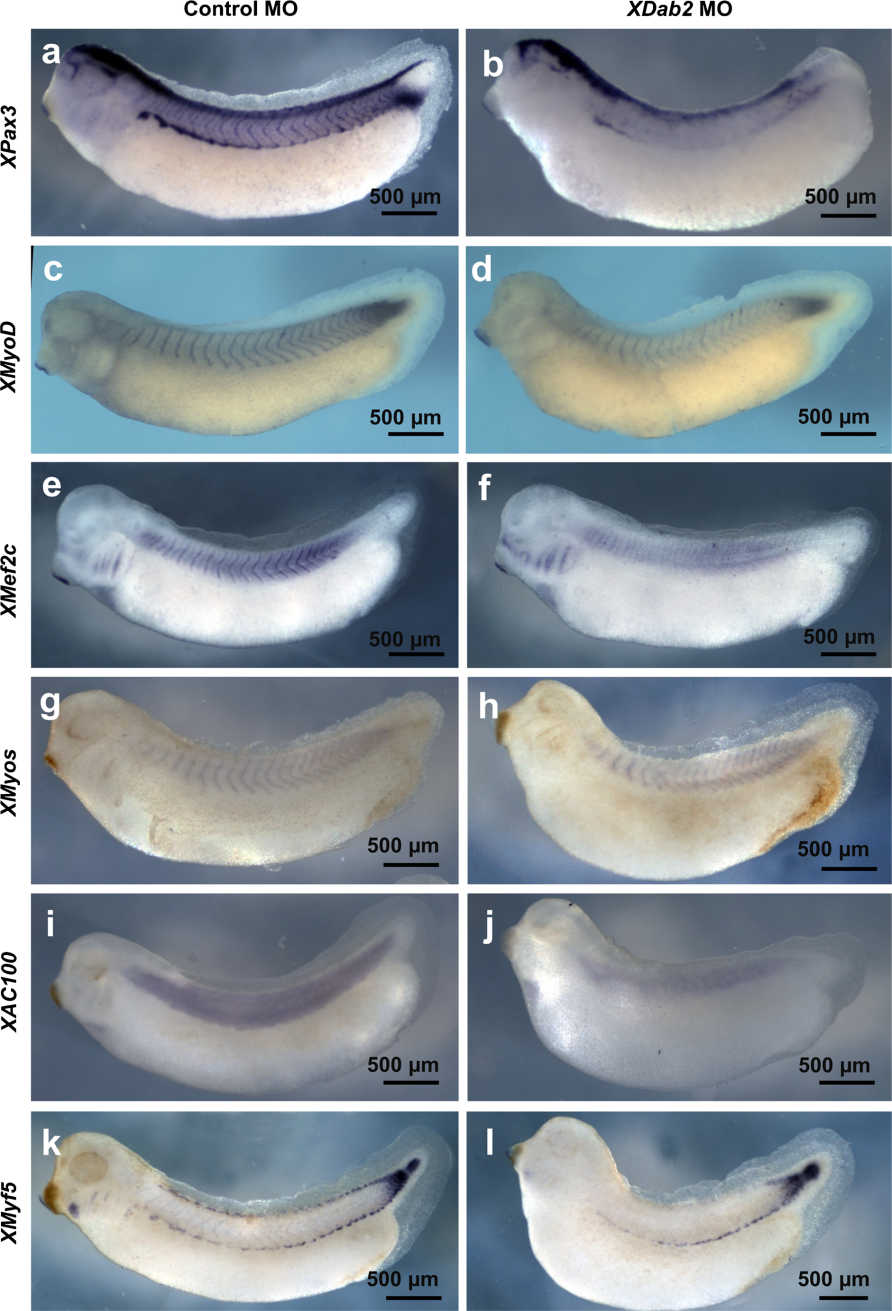
Inhibition of *XDab2* expression by morpholinos resulted in the downregulation of the expression of myogenic transcription factors in somites. Both blastomeres at the 2-cell stage were injected with control MO (**a**, **c**, **e**, **g**, **i**, **k**) or *XDab2* MO (**b**, **d**, **f**, **h**, **j**, **l**), and the injected *Xenopus* embryos were fixed at stages 31–32 for the examination of the expression of *XPax3* (**a**, **b**), *XMyoD* (**c**, **d**), *XMef2c* (**e**, **f**), *XMyos* (**g**, **h**), *XAC100* (**i**, **j**), and *XMyf5* (**k**, **l**) after whole-mount in situ hybridization

Both blastomeres of embryos at the two-cell stage were injected with control MO (Control) or *XDab2* (*XDab2*-MO1 and *XDab2*-MO2) MOs, and the expression of various myogenic and somite markers was examined at stages 31–32 after whole-mount in situ hybridization (Fig. 9). The expression of *XPax3* (Fig. 9a, b), *XMyoD* (Fig. 9c, d), *XMef2c* (Fig. 9e, f), *XMyos* (Fig. 9g, h), *XAC100* (Fig. 9i, j), and *XMyf5* (Fig. 9k, l) in myotomes was downregulated in the *XDab2* MO-injected group as compared with the control MO-injected group. Taken together, these results suggested that *XDab2* was essential for the expression of early myogenic transcription factors, thus implicating that *XDab2* might also be important for early myogenesis in somites during the early development of *Xenopus* embryos.

## Discussion

In this study, we examined the role of Dab2 in the early myogenesis using C2C12 myoblasts in vitro and *Xenopus laevis* embryos in vivo. Results from both qPCR and western blotting showed that when C2C12 cells were induced to undergo myogenic differentiation in a culture medium with a low concentration of serum, Dab2 expression was upregulated in the early stages of myogenic differentiation (i.e., day 1 to day 2 after induction of differentiation). This observation is in line with the former microarray study in which the expression of *Dab2* was upregulated upon treatment of a differentiation medium (Tomczak et al. 2004). However, Dab2 was found to be gradually downregulated in the later stages of myogenic differentiation (i.e., day 3 to day 5). These findings were also in agreement with our previous study with immunohistochemical localization which showed downregulated expression of Dab2 in differentiated skeletal muscles (unpublished data), implying that Dab2 is more important in the early stages of myogenic differentiation.

With gain- and loss-of-function approaches, we established four independent and complementary lines of evidence to support the notion that *Dab2* is a positive regulator of myogenic differentiation. First, the inhibition of *Dab2* expression by miRNA in C2C12 myoblasts in vitro led to a reduction of myotube formation and a decrease of the myogenic fusion index. Second, the upregulated expression of *Dab2* in myoblasts resulted in a substantial increase in the myotube formation and the myogenic fusion index. Third, in myoblasts with stable knockdown of *Dab2* after lentiviral infection of shRNA (clone 5-2), remarkably fewer myotubes and a lower myogenic fusion index were observed. These decreases were further confirmed by western blotting where reduced expression of the myotube marker MHC was found in clone 5-2 cells with *Dab2* knockdown as compared to control C2C12 cells. Fourth and most importantly, re-expression of *Dab2* in clone 5-2 rescued the phenotype and restored the myotube formation to almost the same level of control C2C12 cells. Interestingly, the forced expression of human *DAB2* in clone 5-2 could also restore the normal formation of myotubes. All these observations demonstrated clearly that Dab2 is a positive regulator of early myogenic differentiation in myoblasts.

Among the proteins encoded by *Dab2*, p96 and p67 are the two main isoforms, and p96 has been most studied. The spliced form p67 has been shown to be less efficient in endocytosis (Maurer and Cooper 2005) as it is unable to associate with clathrin and adaptor protein-2 (AP-2) (Morris and Cooper 2001). Besides, due to its small molecular size, p67 has been found to enter the nucleus and acts as a transcription factor in murine embryonal carcinoma cells (Cho et al. 2000). However, both p96 and p67 share the phosphotyrosine-binding (PTB) domain, Asn-Pro-Phe (NPF) motifs and a proline-rich domain (PRD) (Tao et al. 2016). In the present study, overexpression of p96 or p67 was found to enhance myotube formation of C2C12 cells. Thus, these shared domains and motifs might probably play roles in the Dab2-modulated myogenic differentiation, and mutagenesis of Dab2 by domain deletion may help to interrogate the protein–protein interaction during myogenic differentiation in further studies.

Whole-genome microarray profiling of gene expression in differentiating C2C12 myoblasts in vitro identified differentially expressed genes in *Dab2*-knockdown C2C12 cells as compared with their control C2C12 counterpart with normal expression of *Dab2*. A total of 235 probe sets representing 155 genes showed significant changes (*p* < 0.05) of 2-folds or more in their expression in *Dab2*-knockdown myoblasts which also showed a reduced ability to form myotubes. Concomitantly, many genes involved in the early myogenesis were also found to be downregulated. Among them, 127 genes exhibited significant changes of expression (≥ 2-folds). They included *Lama2*, *MyoZ2*, *Myh3*, *Mylk4*, *Tnnc2*, *Actn2*, *Myl4*, *Myl9*, etc. (Supplementary Tables 4 and 5). *Lama2* (laminin alpha 2) is one of the components of the extracellular matrix around muscle fibers (Miyagoe-Suzuki et al. 2000). *Lama2* was first identified as a candidate gene for congenital muscular dystrophy by Helbling-Leclerc et al. (1995). *Dab2* has been found to be a negative regulator of the Wnt/beta-catenin signaling pathway (Jiang et al. 2008, 2009; Zhao et al. 2008). Interestingly, the expression of Wnt/beta-catenin signaling inhibitors *Sfrp1* and *Sfrp2* (Deb et al. 2008; Wang et al. 2008b; Alfaro et al. 2010) was found to be upregulated in our microarray profiling (Supplementary Table 5), and the upregulated expression of *Sfrp2* was verified by qPCR (Supplementary Fig. 1). Previous findings have also shown that Sfrp1 (secreted Frizzled-related protein 1) and Sfrp2 (secreted Frizzled-related protein 2) are inhibitors of myoblast differentiation (Descamps et al. 2008). Therefore, the inhibition of myoblast differentiation in the *Dab2*-knockdown myoblasts might be related to the increased expression of *Sfrp1* and *Sfrp2*. However, how *Dab2* knockdown induces the expression of *Sfrp1* and *Sfrp2* is still unknown. Dab2 may also act in concert with Sfrp2 to negatively regulate Wnt/beta-catenin signaling, but the molecular mechanism underlying the inhibition of myotube formation after *Dab2* knockdown awaits further investigations.

In the present study, differentially expressed genes were found to be significantly associated with the development and functions of the muscular system. The affected genes following *Dab2* knockdown have been correlated with the contraction of striated muscle, differentiation of muscle precursor cells, development of muscle, and other muscular activities (Supplementary Table 6). Network analysis revealed possible functional relationship among genes whose expression levels were altered after *Dab2* knockdown. In this network, *Mef2c* seemed to be an important hub. Its expression was significantly decreased by about 3.66-folds in *Dab2*-knockdown myoblasts. It has been known that Mef2c is an important and essential transcription factor regulating myogenic differentiation (Black and Olson 1998; Wang et al. 2001; Dodou et al. 2003; de Angelis et al. 2005; Haberland et al. 2007). Loss-of-function mutation of the *Mef2* gene in *Drosophila* resulted in differentiation defects of all muscle types (Bour et al. 1995; Lilly et al. 1995). The present study showed that the phosphorylated form of p38 MAPK, which is also the active form of p38 MAPK, was decreased after FGF treatment in *Dab2*-knockdown C2C12 cells. p38 MAPK can phosphorylate Mef2c directly and enhance its transcriptional activity (Han et al. 1997; Wu et al. 2000; Zhao et al. 1999). By acting through Mef2C, p38 MAPK has been found to be crucial to the normal differentiation of C2C12 myoblasts. Thus, *Mef2c* may be plausibly affected by the p38 MAPK signaling pathway in the myogenic differentiation of C2C12 cells although more studies need to be performed to confirm this. Moreover, *Mef2c* was the most affected myogenic transcription factor by *Dab2* knockdown in our microarray analysis. Mef2c is therefore a potentially important molecule along the signaling pathway through which Dab2 regulates the early muscle differentiation.

*Xenopus laevis* has been widely used as a model organism in developmental biology, partly because the whole developmental process from zygote to tadpole can be easily observed and followed. Studies of early embryonic gene functions in *Xenopus* have contributed enormously to elucidating the molecular properties and functional roles of mammalian genes (Zhao et al. 2008). For instance, in *Xenopus laevis* somitogenesis, it has been found that somitic blocks undergo coordinated movements leading to their detachment from the rest of the mesodermal ridge, which is followed by a 90° rotation of the entire metamere, resulting in alignment of the somites parallel to the notochord (Hamilton 1969; Kielbowna 1981). In the present study, by using whole-mount in situ hybridization combined with vibratome sectioning, we found that the expression of *XDab2* was localized to the myotome of somites in *Xenopus* embryos from stages 18 to 35, where genes involved in the early myogenesis such as *XPax3*, *XMyod*, *XMef2c*, and *XMyos* were also expressed. The expression of *XDab2* in somites was decreased from stage 35 with its expression becoming stronger in the intersomitic vasculature. Previous reports have shown that knockdown of *XDab2* expression by antisense morpholinos affected the angiogenesis of *Xenopus* embryos (Cheong et al. 2006), and the injection of *Dab2* mRNA into the one-cell embryo of zebrafish affected the dorsal–ventral patterning (Jiang et al. 2009). Here, we showed that *XDab2* knockdown affected the normal expression of myogenic transcription factors and muscle markers in somites which give rise to early myoblasts. Taken together, it is suggested that *XDab2* positively regulates the early muscle development in *Xenopus* embryos.

In summary, the present study is the first to provide solid evidence that Dab2 is a positive regulator of early myogenesis through regulation of *Mef2c* expression in vitro*.* Moreover, our findings in *Xenopus* embryos also support that Dab2 is a critical regulator of muscle development. This study does not only unveil the positive role of Dab2 in the early myogenesis but also provides novel insight into the molecular mechanism leading to the reduction of the myotube formation when *Dab2* expression is decreased during the early muscle development.

## Electronic supplementary material

ESM 1 (PDF 193 kb).
