# Supplementary material for: Disabled-2: a positive regulator of the early differentiation of myoblasts
Source: Cell Tissue Res. 2020 Jun 30;381(3):493–508. doi: 10.1007/s00441-020-03237-2 (PMC7431403; doi:10.1007/s00441-020-03237-2)
Supplement: Supplementary file 1 — (PDF 193 kb). [file 441_2020_3237_MOESM1_ESM.pdf]

Table S1

Supplementary Table 1 Antibodies

Antigen	Supplier	Dilution
Dab2	BD (610465)	1:500 immunofluorescent staining;<br>1:1500 western blotting
MHC	Developmental studies Hybridoma Bank	1:500 immunofluorescent staining;
p38 MAPK	Cell signaling Technology (4511S)	1:2000 Western blotting;
Phospho-p38 MAPK	Cell signaling Technology (4511P)	1:2000 Western blotting;
GAPDH	Sigma	1:2000 Western blotting;
Alex Fluor-conjugated secondary antibody	Invitrogen<br>(A21202, A31570, A21206, A31572)	1:300 immunofluorescence staining;
HRP-conjugated secondary antibody	Invitrogen<br>(62-6420, 65-6120)	1:8000 (anti-mouse), 1:3000 (anti-rabbit)<br>Western blotting
AP-conjugated anti-Dig	Roche (11093274910)	1:1000 *in sit*u hybridization

Table S2

Supplementary Table 2 Sequences of primers and morpholinos

Propose	Primer name	Primer sequence
Probe preparation	XDab2 96 ISH L	5'-AACCGGATCCCATCCACAACCACAGTCACC-3'
	XDab2 96 ISH R	5'-TCCTCTCGAGCACTTCTCCTGCCTCTTCCA-3'
	XMef2cL	5'-GTGTGGATCCCGCTTGACTGAGGGACTTTC-3'
	XMef2cR	5'-GTGTCTCGAGGGAGCAGAGGGGAAAATCAT-3'
XDab2L cloning	XDab2-96-FW	5’-TTACGGATCCAGGCCTCCAGTCATGTCTAC-3'
	XDab2-96-R1	5’-GCCGATCGATCATTGAGGCTGGCAAGTATC-3'
	Xdab-p96-R2	5’-GCCGATCGATGAGGCTGGCAAGTATCTTC-3'
	XDab2-1L	5’-TGAACATGAACTTCTGCTGATC-3'
	XDab2-2L	5’-TAGCAGCACTGTGTGAACATGA-3'
	XDab2-1R	5’-GGTGACTGTGGTTGTGGATG-3'
XDab2L qPCR	XODC-F	5’-CAGCTAGCTGTGGTGTGG-3'
	XODC-R	5’-CAACATGGAAACTCACACC-3'
	XDab2 RT-L1	5’-GAGGAGCGATGAAAGCAGTC-3'
	XDab2 RT-L2	5’-TGGAAGAGGCAGGAGAAGTG-3'
	XDab2 RT-R1	5’-GCTGCTGAAGTGAGACCTGA-3'
Expression plasmid preparation	XDab2p96-flag-L	5’-GCGCGATATCGGTGTTCCTCTGTCATCTC-3'
	XDab2p96-flag-R	5’-TATAGGCGCGCCAGCAAATGGATCCCCGAATGGA-3'
	mMef2c-HA-L	5'-GCGCGGATCCATGGGGAGAAAAAAGATTCAGATT-3'
	mMef2c-HA-R	5'-GCGCCTCGAGTCATGTTGCCCATCCTTCAG-3'
Morpholino oligonucleotides	XDab2-MO1	5’-ATCTGACTCTTATTTAATGATTGTG-3’
	XDab2-MO2	5’-CCTCCAGTCATGTCTACTGATGTAG-3’
	Control MO	5'-CCTCTTACCTCAGTTACAATTTATA-3'
shRNA knockdown	Control	5’-CCGGCAACAAGATGAAGAGCACCAAC TCGAGTTGGTGCTCTTCATCTTGTTGTTTTT-3’
	TRC1	5’-CCGGCCCTTGAAACTTCCAGAATATC TCGAGATATTCTGGAAGTTTCAAGGGTTTTTG-3’
	TRC2	5’-CCGGCTCCAGTTACTTCAACAATAACTC GAGTTATTGTTGAAGTAACTGGAGTTTTTG-3’
	TRC3	5’-CCGGGCCCTAATGACCCTTGATGATCT CGAGATCATCAAGGGTCATTAGGGCTTTTTG-3’
	TRC4	5’-CCGGCTCCAGTTACTTCAACAATAAC TCGAGTTATTGTTGAAGTAACTGGAGTTTTT-3’
	TRC5	5’-CCGGACTATGGCTTTACGTAAATTACT CGAGTAATTTACGTAAAGCCATAGTTTTTG-3’
miRNA knockdown	L1-Top	5’-TGCTGTTAAGGGTAAAGGTAGCCGCTGTTTTGG CCACTGACTGACTGACAGCGGCTATTTACCCTTAA-3’
	L1-Bottom	5’-CCTGTTAAGGGTAAATAGCCGCTGTCAGTCA GTGGCCAAAACAGCGGCTACCTTTACCCTTAAC
	L2-Top	5’-TGCTGAAATGGTGCAGTTAGCTGAGAGTTTT GGCCACTGACTGACTCTCAGCTCTGCACCATTT-3’
	L2-Bottom	5’-TGCTGTATGGAAGCCGTTCTGTTCTCGTTTG GCACTGACTGACGAGAACAGCGGCTTCCATA-3’
	M2-Top	5’-TGCTGTATGGAAGCCGTTCTGTTCTCGTTTTGGCC ACTGACTGACGAGAACAGCGGCTTCCATA-3’
	M2-Bottom	5’-CCTGAAATGGTGCAGAGCTGAGAGTCAGTCA GTGGCCAAAACTCTCAGCTAACTGCACCATTTC-3’
	M3-Top	5’-TGCTGTTGATATGGAAGCCGTTCTGTGTTTTGG CCACTGACTGACACAGAACGTTCCATATCAA-3’
	M3-Bottom	5’ –CCTGTTGATATGGAACGTTCTGTGTCAGTCAG TGGCCAAAACACAGAACGGCTTCCATATCAAC-3’

Table S3

Supplementary Table 3 Primers for qPCR

Gene Symbol	Primer Name	Primer sequence
Dcn	Dcn-L	5'-GGCTTCTGGCATAATCCCTT-3'
	Dcn-R	5'-CTAGCAAGGTTGTGTCGGGT-3'
Cdh11	Cdh11-L	5'-GACTTGTGAATGGGACTCGG-3'
	Cdh11-R	5'-GTCTTCAAAGGGCCACAAAG-3'
Chrdl1	Chrdl1	5'-GTGCTGCAAGGTGTGCCCAGA-3'
	Chrdl1	5'-TGGTCTCTCGGTCTCCAGTGCT-3'
Lama2	Lama2-L	5'-TCCTCTTGCTCCTGCTCTTG-3'
	Lama2-R	5'-CCCACATGTAGCATTGGTTG-3'
Igf2	Igf2-L	5'-CAGCGGCCTCCTTACCCAACT-3'
	Igf2-R	5'-AAGCTCCCCTCCGCACAGAGT-3'
Ar	Ar-L	5'-GTGAAATGGGACCTTGGATG-3'
	Ar-R	5'-AGCCAGAAGCTTCATCTCCA-3'
Mef2c	Mef2c-L	5'-CCCTCAGTCAGTTGGGAGCTTGC-3'
	Mef2c-R	5'-TGGCGCGTGGTGTGTTGTGG-3'
Pdgfd	Pdgfd-L	5'-AATGCCAACCTCAGGAGAGA-3'
	Pdgfd-R	5'-AAGCAGGTTCCTTGGGTAGC-3'
Myoz2	Myoz2-L	5'-CCCATTTCAGTAATCGTGGG-3'
	Myoz2-R	5'-CCAGGTTGCTTCCATCAACT-3'
Actn2	Actn2	5'-AATCAGATAGAGCCCGGCGTGCAGT-3'
	Actn2	5'-GTGTGAGTTGCACCAGGCAGTGA-3'
Dusp22	Dusp22-L	5'-CTCAACCCTGCCTCCTCTCACC-3'
	Dusp22-R	5'-ACACCAGGCACACCAGCACAT-3'
Myl9	Myl9	5'-GCAACAGCGCCGAGGACTTTTC-3'
	Myl9	5'-TTGGACGTAGCCCTCTGGGG-3'
Myh3	Myh3-L	5'-ACTGTGGAGACAGAAGACAGCAGG-3'
	Myh3-R	5'-GGCTCGTTCAGGTGGGTCAGC-3'
Fyn	Fyn-L	5'-AGCATTACTCAGAGAGAGCCGCA-3'
	Fyn-R	5'-CAACTGCAGGGATTCTCGAGGG-3'
Itga6	Itga6-L	5'-GGAGCCTCTTCGGCTTCT-3'
	Itga6-R	5'-GTACAGGCCCCCTGTTCTGT-3'
Cdh18	Cdh18-L	5'-GCCTTCCTGTCCTCCGCTGGT-3'
	Cdh18-R	5'-CTCTCGCACATCCTCCTCGGAA-3'
Hspb1	Hspb1-L	5'-CAGTGAAGACCAAGGAAGGC-3'
	Hspb1-R	5'-CAGGGGATAGGGAAGAGGAC-3'
Dusp1	Dusp1-L	5'-CGGGAGGAAAGCGCGGTGAAG-3'
	Dusp1-R	5'-GCCCGTTCTGCGGTTCTCCA-3'
Dpt	Dpt-L	5'-CTATGCCTGCATGCCTACAC-3'
	Dpt-R	5'-GATTCGAAGTAGCGGCTCTG-3'
Sfrp2	Sfrp2-L	5'-CGACATCATGGAAACCCTTT-3'
	Sfrp2-R	5'-ACGCCGTTCAGCTTGTAAAT-3'
Gapdh	Gapdh-L	5'-CGTCCCGTAGACAAAATGGT-3'
	Gapdh-R	5'-TTGATGGCAACAATCTCCAC-3'

Table S4

Supplementary Table 4 Genes with expression levels decreased by 2 folds or more in clone 5-2 as compared to control C2C12 myoblasts (p<0.05)

Transcript ID	Gene Symbol	RefSeq	Description	Fold-Change	p-value
10365974	Dcn	NM_007833	decorin	-26.12	0.0009
10581013	Cdh11	NM_009866	cadherin 11	-15.10	0.0001
10483353	Scn7a	NM_009135	sodium channel, voltage-gated, type VII, alpha	-10.30	0.0000
10607124	Chrdl1	NM_001114385	chordin-like 1	-10.19	0.0023
10530854	Tecrl	NM_153801	trans-2,3-enoyl-CoA reductase-like	-8.06	0.0069
10485624	Prrg4	NM_178695	proline rich Gla (G-carboxyglutamic acid) 4 (transmembrane)	-7.72	0.0031
10416215	Loxl2	NM_033325	lysyl oxidase-like 2	-7.06	0.0017
10424119	Nov	NM_010930	nephroblastoma overexpressed gene	-6.47	0.0235
10547153	Alox5	NM_009662	arachidonate 5-lipoxygenase	-6.36	0.0029
10521759	Slit2	NM_178804	slit homolog 2 (Drosophila)	-6.16	0.0051
10429568	Ly6c1	NM_010741	lymphocyte antigen 6 complex,locus C1	-5.89	0.0051
10536334	Dync1i1	NM_010063	dynein cytoplasmic 1 intermediate chain 1	-5.66	0.0012
10553477	Ano5	NM_177694	anoctamin 5	-5.65	0.0017
10358339	Cfh	NM_009888	complement component factor h	-5.30	0.0104
10498907	Glrb	NM_010298	glycine receptor, beta subunit	-5.29	0.0002
10429573	Ly6c2	NM_001099217	lymphocyte antigen 6 complex, locus C2	-5.09	0.0045
10598626	Tspan7	NM_019634	tetraspanin 7	-4.64	0.0003
10540401	Lrrn1	NM_008516	leucine rich repeat protein 1, neuronal	-4.64	0.0276
10368409	Lama2	NM_008481	laminin, alpha 2	-4.59	0.0003
10385513	9930111J21Rik2	NM_173434	RIKEN cDNA 9930111J21 gene 2	-4.55	0.0178
10569344	Igf2	NM_001122737	insulin-like growth factor 2	-4.18	0.0066
10423358	11-Mar	NM_177597	membrane-associated ring finger (C3HC4) 11	-4.12	0.0073
10429564	Ly6a	NM_010738	lymphocyte antigen 6 complex, locus A	-4.07	0.0062
10347277	Igfbp2	NM_008342	insulin-like growth factor binding protein 2	-3.97	0.0471
10600901	Ar	NM_013476	androgen receptor	-3.79	0.0028
10466712	Mamdc2	NM_174857	MAM domain containing 2	-3.75	0.0009
10404439	Serpinb9b	NM_011452	serine (or cysteine) peptidase inhibitor, clade B, member 9b	-3.69	0.0003
10406434	Mef2c	NM_001170537	myocyte enhancer factor 2C	-3.66	0.0291
10606837	Nxf3	NM_001024141	nuclear RNA export factor 3	-3.62	0.0031
10583809	Cnn1	NM_009922	calponin 1	-3.55	0.0226
10583021	Pdgfd	NM_027924	platelet-derived growth factor, D polypeptide	-3.51	0.0035
10501895	Myoz2	NM_021503	myozenin 2	-3.50	0.0362
10422728	Dab2	NM_023118	disabled homolog 2 (Drosophila)	-3.48	0.0023
10466800	Pgm5	NM_175013	phosphoglucomutase 5	-3.46	0.0094
10605181	Renbp	NM_023132	renin binding protein	-3.38	0.0004
10416175	Nefl	NM_010910	neurofilament, light polypeptide	-3.37	0.0110
10581266	Tppp3	NM_026481	tubulin polymerization-promoting protein family member 3	-3.32	0.0055
10578989	Psd3	NM_177698	pleckstrin and Sec7 domain containing 3	-3.26	0.0007
10408543	Mylk4	NM_001166030	myosin light chain kinase family, member 4	-3.25	0.0071
10489545	Tnnc2	NM_009394	troponin C2, fast	-3.25	0.0342
10554667	Tmc3	NM_177695	transmembrane channel-like gene family 3	-3.20	0.0006
10453759	Gm10554	ENSMUST00000097674	predicted gene 10554	-3.19	0.0240
10495878	Ndst4	NM_022565	N-deacetylase/N-sulfotransferase (heparin glucosaminyl) 4	-3.10	0.0039
10430660	Pdgfb	NM_011057	platelet derived growth factor, B polypeptide	-3.08	0.0431
10606016	Il2rg	NM_013563	interleukin 2 receptor, gamma chain	-3.07	0.0014
10569341	H19	NR_001592	H19 fetal liver mRNA	-3.03	0.0205
10407742	Actn2	NM_033268	actinin alpha 2	-3.02	0.0246
10404380	Dusp22	NM_001037955	dual specificity phosphatase 22	-2.97	0.0021
10453747	Colec12	NM_130449	collectin sub-family member 12	-2.97	0.0081

Table S4

Supplementary Table 4   continued

Transcript ID	Gene Symbol	RefSeq	Description	Fold-Change	p-value
10494548	Gja5	NM_008121	gap junction protein, alpha 5	-2.96	0.0266
10505489	Pappa	NM_021362	pregnancy-associated plasma protein A	-2.89	0.0038
10484463	Serping1	NM_009776	serine (or cysteine) peptidase inhibitor, clade G, member 1	-2.89	0.0003
10381798	Myl4	NM_010858	myosin, light polypeptide 4	-2.89	0.0396
10358668	Hmcn1	NM_001024720	hemicentin 1	-2.88	0.0000
10501063	Cd53	NM_007651	CD53 antigen	-2.88	0.0020
10382106	Gm885	NM_001033435	predicted gene 885	-2.85	0.0401
10606820	1700008I05Rik	NM_027952	RIKEN cDNA 1700008I05 gene	-2.85	0.0076
10458046	D0H4S114	NM_053078	DNA segment, human D4S114	-2.84	0.0224
10602756	Smpx	NM_025357	small muscle protein, X-linked	-2.76	0.0306
10554521	Pde8a	NM_008803	phosphodiesterase 8A	-2.75	0.0020
10595324	Htr1b	NM_010482	5-hydroxytryptamine (serotonin) receptor 1B	-2.72	0.0092
10438603	Igf2bp2	NM_183029	insulin-like growth factor 2 mRNA binding protein 2	-2.70	0.0010
10474096	Lrrc4c	NM_178725	leucine rich repeat containing 4C	-2.70	0.0283
10477920	Myl9	NM_172118	myosin, light polypeptide 9, regulatory	-2.69	0.0365
10347036	Mtap2	NM_001039934	microtubule-associated protein 2	-2.68	0.0001
10348580	Klhl30	NM_027551	kelch-like 30 (Drosophila)	-2.67	0.0144
10406934	Etv1	NM_007960	ets variant gene 1	-2.62	0.0203
10469457	Plxdc2	NM_026162	plexin domain containing 2	-2.62	0.0075
10595070	Fam83b	BC120577	family with sequence similarity 83, member B	-2.60	0.0042
10473125	Itga4	NM_010576	integrin alpha 4	-2.59	0.0003
10354374	Slc40a1	NM_016917	solute carrier family 40 (iron-regulated transporter), member 1	-2.58	0.0043
10407797	Prl2c3	NM_011118	prolactin family 2, subfamily c, member 3	-2.58	0.0237
10605113	L1cam	NM_008478	L1 cell adhesion molecule	-2.57	0.0085
10377018	Myh3	NM_001099635	myosin, heavy polypeptide 3, skeletal muscle, embryonic	-2.56	0.0372
10344897	Sulf1	NM_172294	sulfatase 1	-2.54	0.0010
10594963	Unc13c	NM_001081153	unc-13 homolog C (C. elegans)	-2.51	0.0054
10363455	Pcbd1	NM_025273	pterin 4 alpha carbinolamine dehydratase/dimerization cofactor of	-2.50	0.0056
10348194	Efhd1	NM_028889	EF hand domain containing 1	-2.48	0.0055
10419082	5730469M10Rik	BC056635	RIKEN cDNA 5730469M10 gene	-2.48	0.0219
10362596	Fyn	NM_001122893	Fyn proto-oncogene	-2.47	0.0079
10476021	Sirpa	NM_007547	signal-regulatory protein alpha	-2.46	0.0011
10567010	Dkk3	NM_015814	dickkopf homolog 3 (Xenopus laevis)	-2.44	0.0004
10606792	Nxf7	NM_130888	nuclear RNA export factor 7	-2.43	0.0033
10530998	Tmprss11f	NM_178730	transmembrane protease, serine 11f	-2.41	0.0022
10368675	Marcks	NM_008538	myristoylated alanine rich protein kinase C substrate	-2.40	0.0262
10399973	Hdac9	NM_024124	histone deacetylase 9	-2.37	0.0001
10490302	2810021G02Rik	NM_001162922	RIKEN cDNA 2810021G02 gene	-2.36	0.0307
10542965	Sgce	NM_001130190	sarcoglycan, epsilon	-2.34	0.0195
10472820	Itga6	NM_008397	integrin alpha 6	-2.32	0.0079
10495794	Pde5a	NM_153422	phosphodiesterase 5A, cGMP-specific	-2.31	0.0000
10487011	Gatm	NM_025961	glycine amidinotransferase (L-arginine:glycine amidinotransferase)	-2.31	0.0232
10375137	Kcnmb1	NM_031169	potassium large conductance calcium-activated channel, subfamily	-2.29	0.0007
10528207	Cd36	NM_001159557	CD36 antigen	-2.29	0.0283
10423274	Cdh18	NM_001081299	cadherin 18	-2.28	0.0077
10445338	Enpp5	NM_032003	ectonucleotide pyrophosphatase/phosphodiesterase 5	-2.28	0.0112
10485711	Fibin	NM_026271	fin bud initiation factor homolog (zebrafish)	-2.26	0.0106
10462613	Ifit2	NM_008332	interferon-induced protein with tetratricopeptide repeats 2	-2.25	0.0015
10349401	Gpr39	NM_027677	G protein-coupled receptor 39	-2.24	0.0019
10460947	Pygm	NM_011224	muscle glycogen phosphorylase	-2.22	0.0267

Table S4

Supplementary Table 4 continued

Transcript ID	Gene Symbol	RefSeq	Description	Fold-Change	p-value
10555205	Gdpd5	NM_201352	glycerophosphodiester phosphodiesterase domain containing 5	-2.21	0.0452
10521796	Mir218-1	NR_029798	microRNA 218-1	-2.21	0.0337
10579894	Hhip	NM_020259	Hedgehog-interacting protein	-2.21	0.0332
10566578	Gm8979	NR_030719	very large inducible GTPase 1 pseudogene	-2.20	0.0052
10537509	Mgam	NM_001171003	maltase-glucoamylase	-2.20	0.0071
10388254	Aspa	NM_023113	aspartoacylase	-2.17	0.0295
10571142	Gpr124	NM_054044	G protein-coupled receptor 124	-2.16	0.0099
10493108	Crabp2	NM_007759	cellular retinoic acid binding protein II	-2.15	0.0005
10469046	Phyh	NM_010726	phytanoyl-CoA hydroxylase	-2.13	0.0466
10503659	Epha7	NM_010141	Eph receptor A7	-2.12	0.0063
10559248	Tspan32	NM_020286	tetraspanin 32	-2.11	0.0018
10551852	Clip3	NM_001081114	CAP-GLY domain containing linker protein 3	-2.11	0.0079
10536294	Peg10	NM_130877	paternally expressed 10	-2.10	0.0106
10498337	Clrn1	NM_153384	clarin 1	-2.10	0.0002
10513008	Klf4	NM_010637	Kruppel-like factor 4 (gut)	-2.09	0.0075
10362372	9330159F19Rik	NM_001162537	RIKEN cDNA 9330159F19 gene	-2.08	0.0429
10355259	Myl1	NM_021285	myosin, light polypeptide 1	-2.07	0.0358
10492021	Postn	NM_015784	periostin, osteoblast specific factor	-2.07	0.0414
10528810	Gm10471	NM_001177579	predicted gene 10471	-2.07	0.0115
10407286	BC067074	ENSMUST00000078163	cDNA sequence BC067074	-2.07	0.0295
10592251	Pknox2	NM_001029838	Pbx/knotted 1 homeobox 2	-2.06	0.0186
10401616	Mlh3	NM_175337	mutL homolog 3 (E coli)	-2.06	0.0068
10490972	Trim55	NM_001081281	ripartite motif-containing 55	-2.04	0.0059
10366346	Phlda1	NM_009344	pleckstrin homology-like domain, family A, member 1	-2.04	0.0015
10572130	Lpl	NM_008509	lipoprotein lipase	-2.03	0.0224
10408928	Hspb1	NM_013560	heat shock protein 1	-2.03	0.0102
10496091	Lef1	NM_010703	lymphoid enhancer binding factor 1	-2.02	0.0031
10449284	Dusp1	NM_013642	dual specificity phosphatase 1	-2.00	0.0078

Table S5

Supplementary Table 5 Genes with expression levels increased by 2 folds or more in clone 5-2 as compared to control C2C12 myoblasts (p<0.05)

Transcript ID	Gene Symbol	RefSeq	Description	Fold-Change	p-value
10601588	*3110007F17Rik*	BC027572	RIKEN cDNA 3110007F17 gene	2.07	0.0498
10405047	*Aspn*	NM_025711	asporin	4.42	0.0160
10490913	*Car3*	NM_007606	carbonic anhydrase 3	3.16	0.0093
10362896	*Cd24a*	NM_009846	CD24a antigen	3.24	0.0365
10402473	*Clmn*	NM_053155	calmin	2.22	0.0032
10537051	*Cpa1*	NM_025350	carboxypeptidase A1	2.75	0.0024
10537038	*Cpa5*	NM_144537	carboxypeptidase A5	2.05	0.0010
10523120	*Cxcl5*	NM_009141	chemokine (C-X-C motif) ligand 5	2.48	0.0446
10351293	*Dpt*	NM_019759	dermatopontin	5.96	0.0062
10407072	*Elovl7*	NM_029001	ELOVL family member 7, elongation of long chain fatty acids	2.70	0.0318
10475932	*Fbln7*	NM_024237	fibulin 7	2.21	0.0229
10359571	*Fmo1*	NM_010231	flavin containing monooxygenase 1	2.20	0.0115
10349947	*Fmod*	NM_021355	fibromodulin	2.46	0.0259
10454103	*Gm2889*	XR_032134	predicted gene 2889	2.04	0.0381
10604674	*Gm773*	BC147685	predicted gene 773	2.43	0.0049
10574276	*Gpr97*	NM_173036	G protein-coupled receptor 97	2.13	0.0071
10599348	*Gria3*	NM_016886	glutamate receptor, ionotropic, AMPA3 (alpha 3)	6.46	0.0053
10384223	*Igfbp3*	NM_008343	insulin-like growth factor binding protein 3	4.56	0.0295
10602704	*Klf8*	NM_173780	Kruppel-like factor 8	2.74	0.0136
10438753	*Leprel1*	NM_173379	leprecan-like 1	2.08	0.0150
10542470	*Mgst1*	NM_019946	microsomal glutathione S-transferase 1	2.72	0.0079
10473602	*Olfr1189*	NM_146772	olfactory receptor 1189	2.59	0.0309
10423654	*Osr2*	NM_054049	odd-skipped related 2 (Drosophila)	2.71	0.0052
10543959	*Ptn*	NM_008973	pleiotrophin	2.94	0.0241
10542911	*Samd9l*	NM_010156	sterile alpha motif domain containing 9-like	2.65	0.0089
10570957	*Sfrp1*	NM_013834	secreted frizzled-related protein 1	2.52	0.0498
10492798	*Sfrp2*	NM_009144	secreted frizzled-related protein 2	7.60	0.0256
10489759	*Sulf2*	NM_028072	sulfatase 2	2.24	0.0102

Table S6

Supplementary Table 6 Functional correlation of differentially expressed in clone 5-2 with the development and/or functions of the muscular system ($p<0.05$)

Functions Annotation	p-Value	Molecules
muscle contraction	8.99E-06	ACTN2,CNN1,CTGF,GJA5,MYH1,MYL4,RYR3,SCN7A,SMPX, TNNC2,TRDN
contraction of striated muscle	3.06E-05	CTGF,MYH1,MYL4,RYR3,SMPX,TNNC2
differentiation of muscle precursor cells	6.92E-05	DCN,IGF2,IGFBP3,ITGA6,NDN,Prl2c2 (includes others)
development of muscle	1.03E-04	AR,DUSP1,ETV1,HDAC9,IGF2,LAMA2,MEF2C,MYH3,NOV,P APPA,PDGFB,SGCE
differentiation of muscle cells	2.25E-04	DCN,IGF2,IGFBP3,ITGA6,LAMA2,MEF2C,NDN,PDE5A,Prl2c2 (includes others)
formation of myotube	4.13E-04	IGF2,LAMA2,NOV,PAPPA
contractility of smooth muscle	5.08E-04	SULF1,SULF2
differentiation of myoblasts	5.85E-04	IGF2,IGFBP3,ITGA6,NDN,Prl2c2 (includes others)
migration of smooth muscle cells	2.97E-03	DUSP1,F3,IGFBP2,NOV,PDGFB
fusion of myotube	3.67E-03	AR,IGF2
growth of muscle cells	4.29E-03	DUSP1,KLF4,PAPPA,PDGFB
differentiation of vascular smooth muscle cells	4.46E-03	DCN,MEF2C
myogenesis	5.06E-03	AR,DUSP1,IGF2,LAMA2,PDGFB
differentiation of skeletal muscle cells	8.31E-03	IGF2,MEF2C
angiogenesis of growth plate	9.29E-03	CTGF
arrest in G2/M phase transition of muscle cell lines	9.29E-03	IGF2
endochondral ossification of growth plate	9.29E-03	CTGF
force generation of extensor digitorum longus muscle	9.29E-03	AR

Table S7

Supplementary Table 7 Changes of expression levels of myogenic transcription factors in clone 5-2 as compared to control C2C12 myoblasts

Gene Symbol	RefSeq	p-value	Fold-Change
Mef2c	NM_001170537	0.029084	-3.65868
Myf5	NM_008656	0.566123	-1.10401
Pax7	NM_011039	0.803499	1.04651
Myod1	NM_010866	0.977681	-1.01153
Myf6	NM_008657	0.353469	1.10489
Myog	NM_031189	0.937721	-1.02294
Pax3	NM_008781	0.569902	-1.05367

Fig. S1

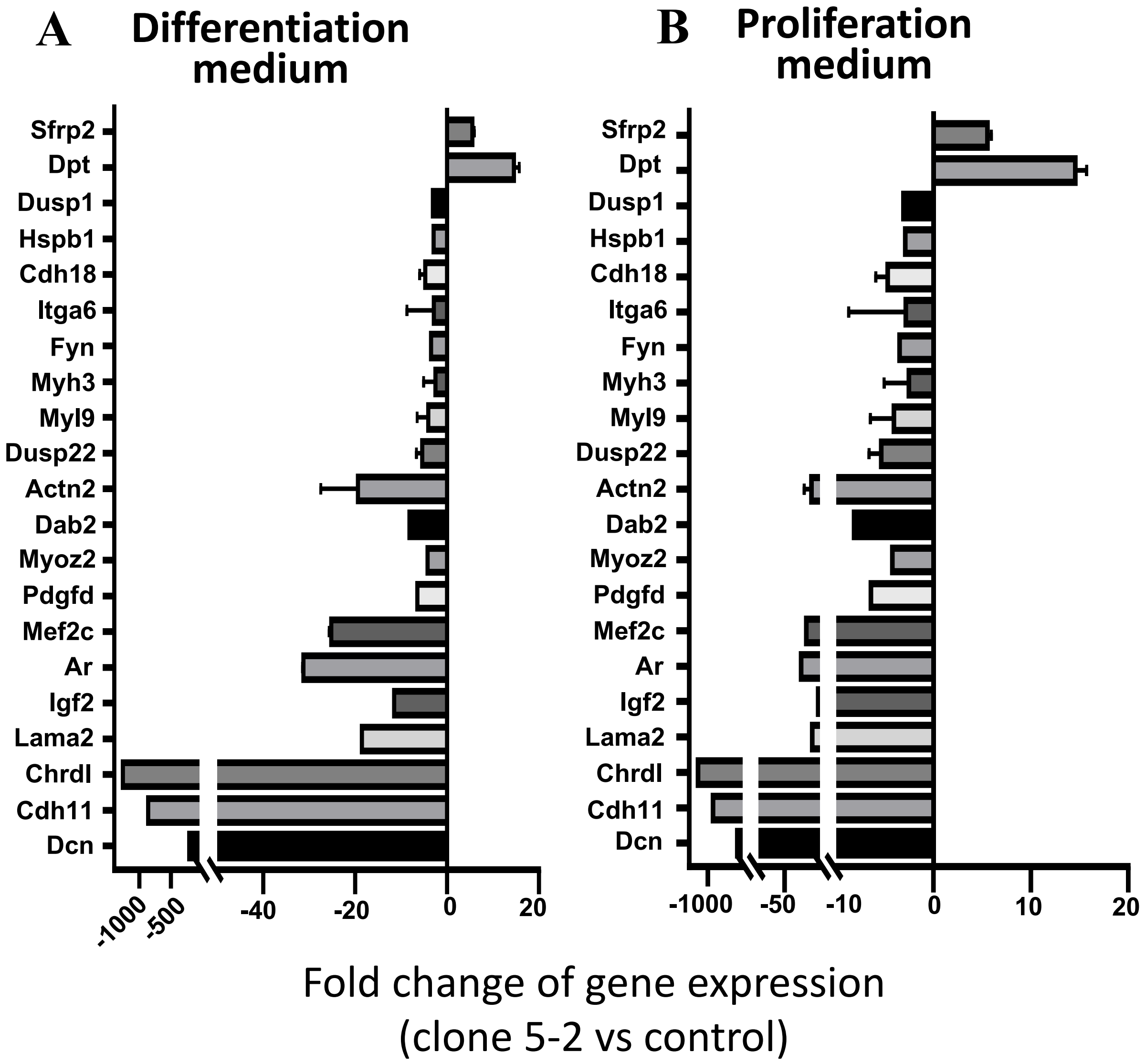

Supplementary Fig. 1. Relative expression of differentially expressed gene in clone 5-2 comparing to control C2C12 myoblasts.

Clone 5-2 cells and C2C12 myoblasts were cultured in the differentiation (A) or proliferation medium (B) for 2 days prior to qPCR. Results show the relative expression of some of the differentially expressed genes displayed in the microarray. Fold changes are calculated based on the expression in clone 5-2 cells relative to that in C2C12 myoblasts, and presented as mean±S.D. Expression levels are normalized against the expression of *Gapdh*.
